# Single-molecule analysis of DNA base-stacking energetics using patterned DNA nanostructures

**DOI:** 10.1038/s41565-023-01485-1

**Published:** 2023-08-17

**Authors:** Abhinav Banerjee, Micky Anand, Simanta Kalita, Mahipal Ganji

**Affiliations:** 1 Department of Biochemistry, Indian Institute of Science, Bangalore, India; 2 New Chemistry Unit and Chemistry and Physics of Materials Unit, The Jawaharlal Nehru Centre for Advanced Scientific Research, Bengaluru, India

**Keywords:** Nanobiotechnology, Biopolymers, Nanobiotechnology, Organizing materials with DNA, DNA nanostructures

## Abstract

The DNA double helix structure is stabilized by base-pairing and base-stacking interactions. However, a comprehensive understanding of dinucleotide base-stacking energetics is lacking. Here we combined multiplexed DNA-based point accumulation in nanoscale topography (DNA-PAINT) imaging with designer DNA nanostructures and measured the free energy of dinucleotide base stacking at the single-molecule level. Multiplexed imaging enabled us to extract the binding kinetics of an imager strand with and without additional dinucleotide stacking interactions. The DNA-PAINT data showed that a single additional dinucleotide base stacking results in up to 250-fold stabilization for the DNA duplex nanostructure. We found that the dinucleotide base-stacking energies vary from −0.95 ± 0.12 kcal mol^−1^ to −3.22 ± 0.04 kcal mol^−1^ for C|T and A|C base-stackings, respectively. We demonstrate the application of base-stacking energetics in designing DNA-PAINT probes for multiplexed super-resolution imaging, and efficient assembly of higher-order DNA nanostructures. Our results will aid in designing functional DNA nanostructures, and DNA and RNA aptamers, and facilitate better predictions of the local DNA structure.

## Abstract (web summary)

DNA-based point accumulation in nanoscale topography (DNA-PAINT) super-resolution imaging reveals the kinetics of the free energy of base-stacking interactions of all 16 dinucleotide combinations at the single-molecule level in patterned DNA-origami nanostructures.

## Main

DNA undergoes constant deformations for cellular needs, yet is efficiently transferred through many generations. This calls for robust local interactions to ensure long-term stability. The thermodynamic stability is achieved by base-pairing^1^ and base-stacking^2^ interactions. Biochemical analyses suggest that base-stacking energies predominantly contribute to the stabilization of DNA compared with the base-pairing interactions^3^. The base-stacking interactions play a role in nucleic acids metabolic processes^2,4,5^, as well as aid in designing hierarchical DNA nanostructures and aptamers^6–8^.

DNA nanotechnology in recent decades has seen tremendous progress in generating a variety of functional nanostructures, finding applications in diverse disciplines^9–12^. These designs mainly rely on the programmability of DNA based on the base pairing of complementary nucleotides. Base-stacking interactions, in addition, have enabled hierarchical assembly of modular and functional nanostructures^6,13–15^. However, the choice of base-stacking interactions utilized for assembling higher-order nanostructures is largely random as the actual strength of individual dinucleotide base-stacking is not known.

So far, base-stacking energetics have been measured from bulk biochemical studies^16–24^. On the basis of these data, a unified nearest-neighbour model has been developed, which predicts the sequence-dependent DNA thermal stability^25^. However, the nearest-neighbour approximation does not separate base-pairing and base-stacking interactions. Earlier attempts to measure base-stacking energies relied on biochemically analysing the relative electrophoresis on urea polyacrylamide gels of nicked or gapped DNA molecules or thermal denaturation^3,26–28^. Recently, single-molecule optical tweezer experiments measured the force-dependent dissociation rate between the blunt ends of parallel DNA beams^29^. This assay estimated free energies between individual base pairs by means of extrapolating the force applied across DNA beams consisting of several blunt DNA ends. However, direct measurement of individual base-stacking forces, especially at the single-molecule level, between dinucleotides was not possible due to the unavailability of sensitive experimental techniques. Other recent parallel studies have conducted single-molecule force spectroscopy experiments to fill the knowledge gap^30,31^, albeit under non-equilibrium conditions^30^.

Here we measure individual dinucleotide base-stacking energetics at the single-molecule level using DNA-based point accumulation in nanoscale topography (DNA-PAINT) while exploiting DNA nanotechnology for multiplexing^32–34^. DNA-PAINT enables us to directly access the binding dynamics of a fluorophore-labelled oligonucleotide (denoted as imager strand) to its complementary strand (denoted as docking strand) positioned on a DNA-origami nanostructure^34,35^. We compare the imager strand’s binding kinetics with stacking and non-stacking configurations at its terminal nucleotide in a single experiment by multiplexed imaging of patterned origami structures. Kinetic analysis of our single-molecule data resulted in an unexpectedly wide range of dwell-time stabilizations due to individual base-stacking energies. On the basis of the observed dwell-time stabilizations, we designed probes for DNA-PAINT imaging and experimentally showed their applicability in simultaneous super-resolution imaging. We also demonstrated the application of stacking energetics for folding higher-order DNA nanostructures.

## Experimental design to measure base-stacking interactions

**Fig. 1:**
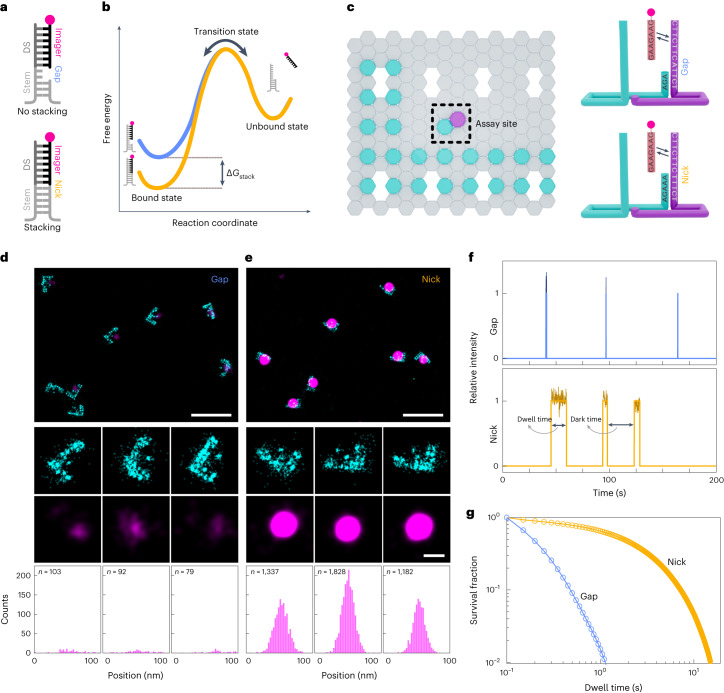
Single-molecule assay for studying dinucleotide base-stacking interactions. **a**, Schematic representation of the two configurations of gap and nick. DS, docking strand. **b**, Representative free-energy diagram of the bound and unbound states. The bound state under the nick configuration would show greater stabilization and thus lower free energy (orange curve) than the bound state under the gap configuration (blue curve). **c**, Graphical representation of the origami layout. Left: the L-shaped grid with cyan-colour extensions for identifying the locations of origami structures, and the assay site with magenta-colour extension for studying base-stacking interactions. Right: detailed view of the assay site where two staples are used together to generate a gap or a nick configuration. **d**,**e**, DNA-PAINT data of origami grid imaged with Atto647N imager (cyan) and assay site imaged with Cy3B imager (magenta). Gap (**d**) and nick (**e**): row 1 shows example large field of views; rows 2 and 3 show individual origami grids and their colocalized assay site, respectively; row 4 shows the histograms of number of bound frames for each assay site. The appearance of higher number of bound frames in the stacked state is because individual binding events persist longer in case of the nick than the gap. **f**, Representative individual assay site time traces indicating dark time and dwell time. Darker shades represent the relative photon counts in each frame across each binding event. **g**, Cumulative survival fraction of events showing equal to or greater than shown dwell time (*n* = 140,649 for gap and *n* = 216,040 for nick). Scale bars, 200 nm (row 1 in **d** and **e**) and 40 nm (rows 2 and 3 in **d** and **e**). Source data

We present a single-molecule assay based on DNA-PAINT^34^ to deduce the base-stacking interactions between any dinucleotides. We designed two configurations with the docking strand extended from a double-stranded DNA duplex (Fig. 1a). In the first design, the imager binding leaves a two-nucleotide gap between the imager’s 5′-nucleotide and the stem’s 3′-nucleotide (Fig. 1a, top). The second configuration carries the same sequence as the first, but lacks the two-nucleotide gap (that is, the nick), facilitating base-stacking interactions between the terminal nucleotides of the imager and stem (Fig. 1a, bottom). These designs were inspired by the ensemble averaging assays that attempted to quantify base-stacking interactions^3,26,36^. We analyse the binding kinetics of imager–docking strand hybridization using speed-optimized DNA-PAINT probes^37,38^ for extracting the free energy of dinucleotide base stacking (Fig. 1b).

## Imager binding dynamics on the gap and nick configurations

**Extended Data Fig. 1:**
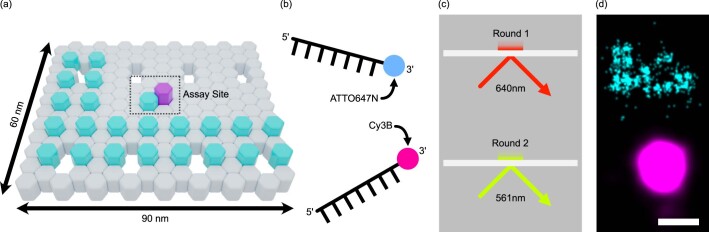
DNA origami design and imaging scheme. **(a)** Graphical layout of the DNA origami design used in the experiments. Cyan hexagons represent the extensions used to image the grid. Magenta hexagon is used to represent extension at the assay site. **(b)** Graphical representation of the two fluorescent imager strands used in the experiments. Imager strand labelled with ATTO647N (top) was used for exciting with the 640 nm laser, and imager labelled with Cy3B (bottom) was used with the 561 nm laser. **(c)** Imaging was performed in TIRF mode in two rounds. First, using the 640 nm laser to excite the ATTO647N fluorophore-labelled imager for imaging the grid (cyan in (a)), and second, using the 561 nm laser to excite the Cy3B fluorophore-labelled imager for imaging assay site (magenta in (a)). **(d)** Example super-resolved images obtained from the two rounds of imaging. (Scale bars: 40 nm (d)).

We designed two rectangular DNA-origami structures^32^ in which the assay site carried the docking strands extended from double-stranded stems in two different configurations (Fig. 1c). The origami structures also carry another set of docking strands in an ‘L’ shape for identifying their locations in the imaging field (henceforth called the grid), enabling us to neglect spurious signals arising from any non-specific imager binding. We imaged these origami structures using two-colour DNA-PAINT under a total internal reflection fluorescence (TIRF) microscope (Fig. 1c and Extended Data Fig. 1)^35,39^.

The reconstructed DNA-PAINT data of the gap and nick configurations showed a distinct number of localizations (Fig. 1d,e and Supplementary Fig. 1a). The nick site appeared brighter, resulting from higher number of bound frames (Fig. 1d,e, bottom). To understand the origins of this difference, we inspected the binding time traces of individual spots. The time traces showed longer dwell times on the nick configuration compared with the gap (Fig. 1f and Supplementary Fig. 1b). Correspondingly, the cumulative survival plot of the imager dwell times showed about tenfold slower decay on the nick configuration (Fig. 1g). These results indicated that an extra dinucleotide base-stacking interaction substantially stabilizes the bound state of the imager, establishing that base-stacking interactions are directly measurable at the single-molecule level.

Measuring absolute, yet weak base-stacking energetics via this experimental approach requires comparing the binding kinetics from two different measurements, making our results sensitive to variations in ionic strength of the buffer, ambient temperature and concentration of imager strands^40^. This is especially relevant for our imager–docking strand hybrid as their melting point is below room temperature.

## Simultaneous measurement of four base-stacking interactions

**Fig. 2:**
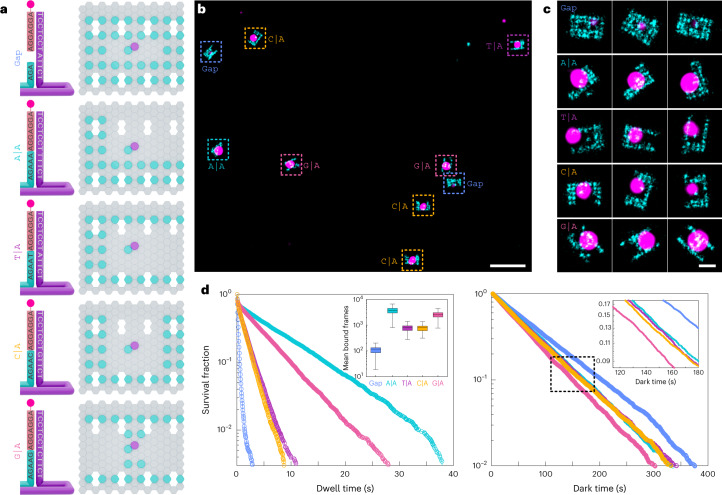
Simultaneous DNA-PAINT imaging of a gap and four base-stacking interactions. **a**, Graphical representation of the five origami grids (right) and the corresponding assay sites (left) used for parallel imaging of four stack combinations possible by a single imager sequence. The stem layouts and sequences are outlined for all the five combinations where the imager stacks on top of the stem (left). Unique grid designs enable parallel imaging (right). **b**, Representative field of view showing all five grid shapes imaged with Atto647N imager and corresponding assay site imaged with Cy3B imager. **c**, Representative picked origami grids and assay site. **d**, Cumulative survival fractions plot showing the fraction of events at indicated dwell times (left) or dark times (right) greater than or equal to the given value (*n* = 8,104, 5,823, 6,860, 8,269 and 9,425 for gap, A|A, T|A, C|A and G|A, respectively). The inset in the left panel shows a box plot with mean number of bound frames per assay site (centre line, median; box limits, upper and lower quartiles; whiskers, 1.5× interquartile range) and the inset in the right panel is the zoomed in view of the rectangular region. Scale bars, 200 nm (**b**) and 40 nm (**c**). Source data

A multiplexed imaging modality was set up to abolish any possible variations in the binding kinetics due to experimental variations. We designed five rectangular DNA-origami structures carrying extensions in unique grid patterns (box, L, U, C and H shapes), thus making them visually distinguishable upon DNA-PAINT imaging (Fig. 2a). Each grid houses an assay site consisting of a unique terminal nucleotide on the stem enabling us to image all five possible interactions (a gap and four nick configurations) simultaneously with a single imager. For example, an imager ending with adenine nucleotide would allow us to experiment with the gap, A|A, T|A, C|A and G|A stacking interactions (Fig. 2a). Here, G|A means 5′-G stacking on 3′-A in a single-stranded DNA of 5′-GA-3′.

We imaged all five origami structures with two-colour DNA-PAINT in successive rounds as mentioned earlier (Extended Data Fig. 1). We maintained a high density of origami distribution allowing us to obtain statistically robust data in a single imaging run. DNA-PAINT super-resolution imaging is indispensable in this scenario as diffraction-limited imaging would not allow us to distinguish binding events occurring on proximally separated origami structures. Reconstructed data show clear, distinguishable origami grids, enabling us to visually identify each gap and nick configuration (Supplementary Fig. 2) that colocalized with a single spot arising from the assay site interactions (Fig. 2b,c). We then extracted the binding kinetics of all five configurations.

Our kinetic analysis revealed that each configuration has a characteristic dwell-time distribution, establishing that the base-stacking interactions are unique to the dinucleotide combinations (Fig. 2d, left). Interestingly, we also observed a considerable increase in the binding frequency at nick configurations compared with the gap, as evident from the dark-time distributions (Fig. 2d, right). These data indicate that the binding strength of the imager is dependent on additional stabilization provided by the stacked dinucleotides.

## Measuring all 16 possible base-stacking energetics

**Fig. 3:**
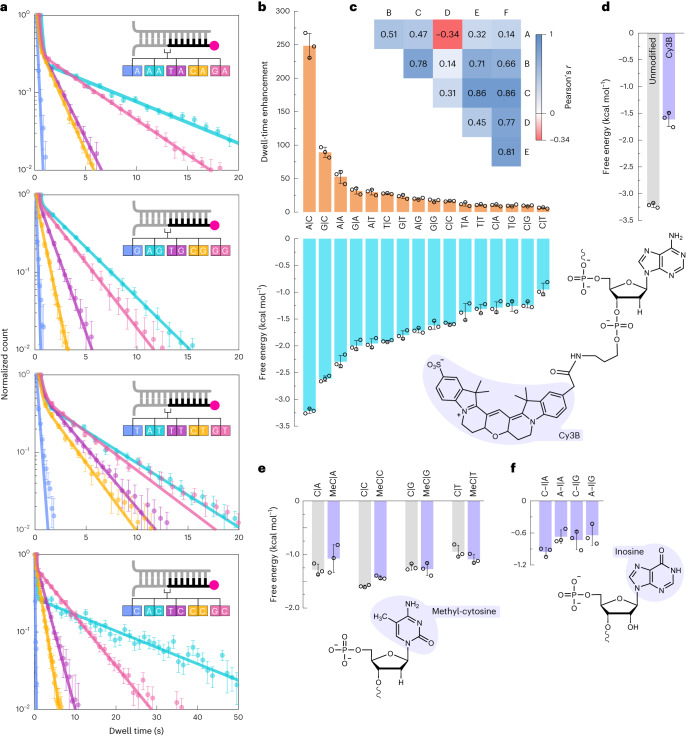
Imaging base-stacking interactions of all 16 possible dinucleotide combinations. **a**, Histograms showing dwell-time distributions (points) and mathematical fits to the data (curves). Each plot, obtained from single simultaneous imaging rounds, shows four base-stacking interactions and a gap as represented in the insets with the corresponding colour combinations. All the gap datasets were fit with a mono-exponential function and nick datasets were fit with a bi-exponential function to obtain the corresponding off-rate constants. Mean and standard deviations were obtained via 100 iterations of bootstrapping. The number of dwell times analysed for all 20 datasets is shown in Extended Data Fig. 2a. **b**, The dwell-time enhancement obtained by taking the ratio of off-rate constants of the gap to that of the nick (top), and the calculated dinucleotide base-stacking free energetics (bottom). **c**, Pearson’s correlation matrix showing correlation between deduced base-stacking energetics (A, ref. ^25^; B, ref. ^29^; C, ref. ^3^; D, ref. ^36^; E, ref. ^28^; F, current study). **d**, Effect of fluorophore-modified adenine on base-stacking energy of A|C. **e**, Effect of methyl-cytosine (MeC) on base-stacking energies. **f**, Base-stacking energies between inosine (I)—paired with either cytosine or adenine—with other nucleotides. Data represent means and standard deviations of three individual datasets (**b** and **d**–**f**). Source data

**Extended Data Fig. 2:**
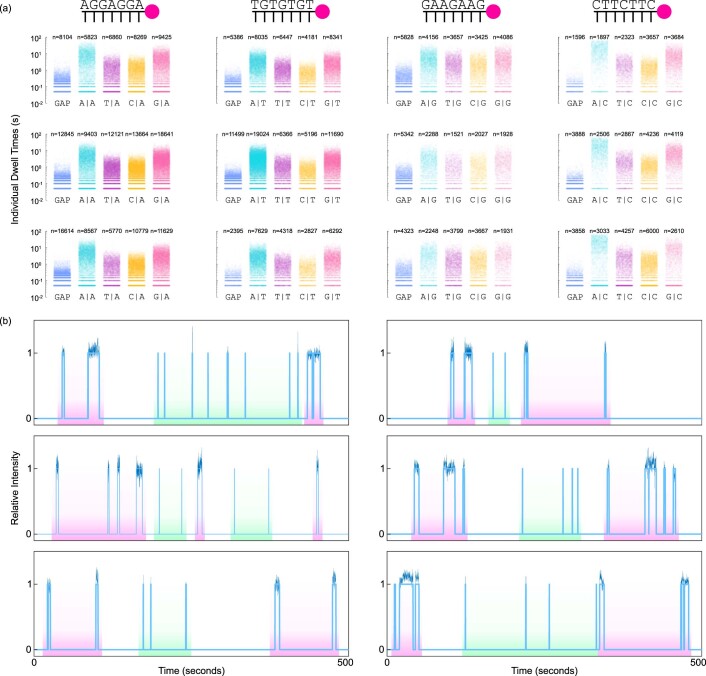
Dwell times analysis measured under different stacking conditions. **(a)** Distribution of individual dwell times plotted over a defined randomized spread for each population. Each spot is plotted with a 97% transparency to graphically represent the density spread of the dwell times. These data are related to Fig. 3a. **(b)** Representative idealized time traces of imager binding overlaid with relative photon count on each bound frame at the assay site showing consecutive short- and long-lived dwell times. Green shade indicates spans of short-lived and magenta shade indicates long-lived dwell times. Source data

**Extended Data Fig. 3:**
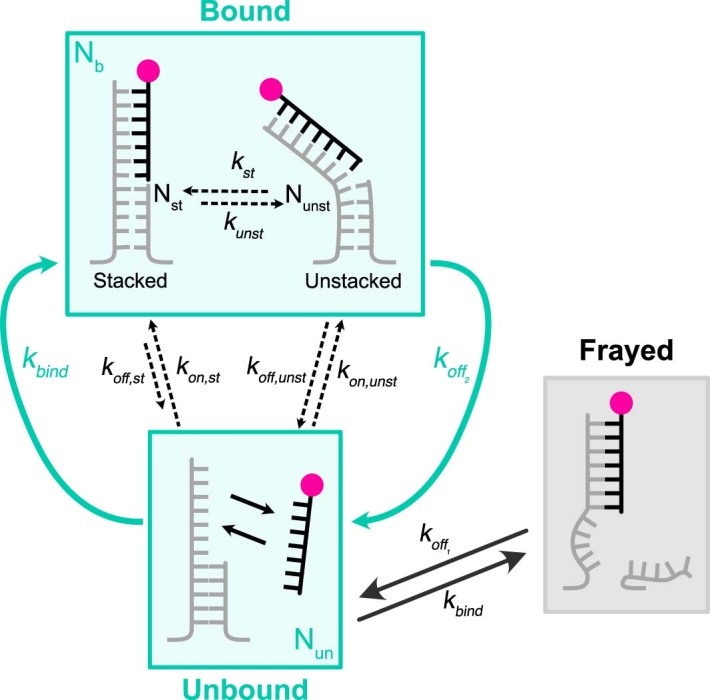
Kinetic model describing the binding dynamics of imager at the nicked assay site In this configuration, the imager binding results in consecutive short-lived and long-lived binding times that we attribute to the phenomenon of stem fraying and reannealing (Supplementary Fig. 4b). The short-lived population exists in a frayed state where the shorter binding events occurred consecutively for a considerable duration, indicating the frayed stem does not reanneal within a binding event. The imager binding rate (k_bind_) would be minimally affected by the stem fraying. The binding to the un-frayed state can be represented as two populations, one with stacking, and one without stacking. These states would transition between each other within a single binding event, giving rise to two populations, N_st_ for stacked states and N_unst_ for the unstacked states. These two states would comprise of the total bound population Nb. For further explanation of the model and derivation of the stacking free energy see methods part.

To deduce all 16 possible dinucleotide base-stacking energies, we designed four separate simultaneous experiments using different imager and docking-strand sequences (insets in Fig. 3a). While the dwell-time distribution of the gap data showed a single population, the base-stacking data showed two clear populations in which shorter dwell times resembled the gap configuration, and the longer ones varied depending on the dinucleotide under investigation (Extended Data Fig. 2a). Interestingly, we observed spans of short- and long-lived binding events (Extended Data Fig. 2b). These data indicate that the imager binds on nick configuration in two different modes, terminal nucleotides stacking or without stacking, equivalent to the gap (Extended Data Fig. 3). The appearance of an unstacked population is probably due to the stem fraying where all five bases undergo melting (Extended Data Fig. 3)^41^. A similar phenomenon has been recently observed at the nick site^42^. This hypothesis was corroborated by the fact that we observed two clear populations in the individual dwell-time distributions (Extended Data Fig. 2). On this basis, we built a kinetic model for both the gap and nick configurations for extracting the rate constants for imager binding (Extended Data Fig. 3 and Methods).

The gap and nick dwell-time distributions were fit with mono- and bi-exponential functions, respectively, to obtain the off-rate constants (Methods, Fig. 3a, Supplementary Fig. 3 and Supplementary Table 1).

**Extended Data Fig. 4:**
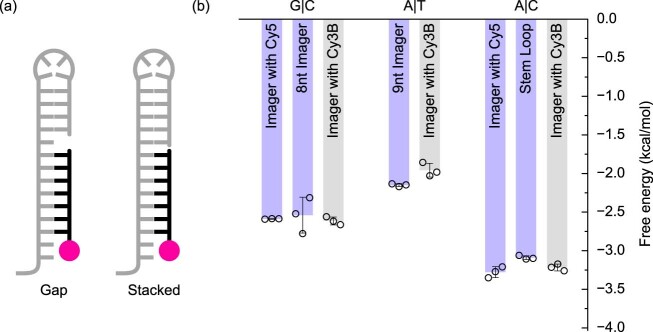
Orthogonal configurations and sequences used to measure base stacking interactions. **(a)** Graphical representation of the orthogonal stem configuration. In this configuration the stem is formed by hairpin formation from the docking strand extension. This configuration carries different stem sequence, providing orthogonal local context in which the stacking energy is measured. This stem is reported to be extremely stable.1 **(b)** Comparative free energy values of stacks measured using orthogonal sequence contexts, designs, and fluorophores. Data represent means and standard deviations of three individual datasets. Source data

We calculated the fractional enhancement in the binding time by taking the ratio of the gap to nick configuration, that is, *k*_off_/*k*_off,2_, which showed a wide distribution depending on the dinucleotide combination, starting from around 6-fold for C|T to 250-fold for A|C (Fig. 3b). We suspected that this unexpectedly large fold change in the binding time was due to the sequence context. We challenged this surprisingly large fractional enhancement for A|C by designing an orthogonal assay site configuration with a different stem sequence. However, we observed similar fractional enhancement in dwell times, validating the accuracy of our approach (Extended Data Fig. 4). In addition, we observed identical fractional enhancement with a different fluorophore on the imager strand, ruling out the possibility of the fluorophore’s intervention in the measured kinetics (Extended Data Fig. 4b and Supplementary Fig. 4d). Although fluorophore photobleaching presents a limitation for single-molecule experiments^43^, we note that the photobleaching rate (*k*_photobleaching_ = 0.0007 s^−1^; Supplementary Fig. 5) is substantially lower than the slowest measured *k*_off,2_ = 0.043 s^−1^ for A|C, indicating that these measurements are unaffected by the fluorophore’s photophysical properties.

## Stacking interactions enhance binding rate

The DNA-PAINT data also provide us with the dark times between the consecutive imager binding events, whose histogram showed a mono-exponential distribution (Supplementary Fig. 6). In most cases, the nick configurations showed a considerably higher binding frequency (*k*_bind_) compared with gaps (Supplementary Fig. 7a and Supplementary Table 2). We anticipate that the nick configuration would mechanistically have two opposing effects on *k*_bind_. First, the imager would experience a steric hindrance by the stem site, hence negatively impacting the initiation of binding resulting in decreased *k*_bind_. Second, base-stacking interaction between the stem and the imager acts as an additional nucleation site for binding, resulting in increased *k*_bind_ (ref. ^44^). Indeed, we observe a positive correlation between the dinucleotide stacks with fractional enhancement in dwell time and enhancement in *k*_bind_ (Supplementary Fig. 7a). In addition, enhancement in the *k*_bind_ negatively correlated with the bulkiness of the underlined stacked dinucleotides that could potentially cause steric hindrance for the imager binding (Supplementary Fig. 7b). The overall increase in the *k*_bind_ is probably because of base-stacking interactions outcompeting the steric hindrance^44^.

## Calculating stacking free energy from binding kinetics

**Extended Data Fig. 5:**
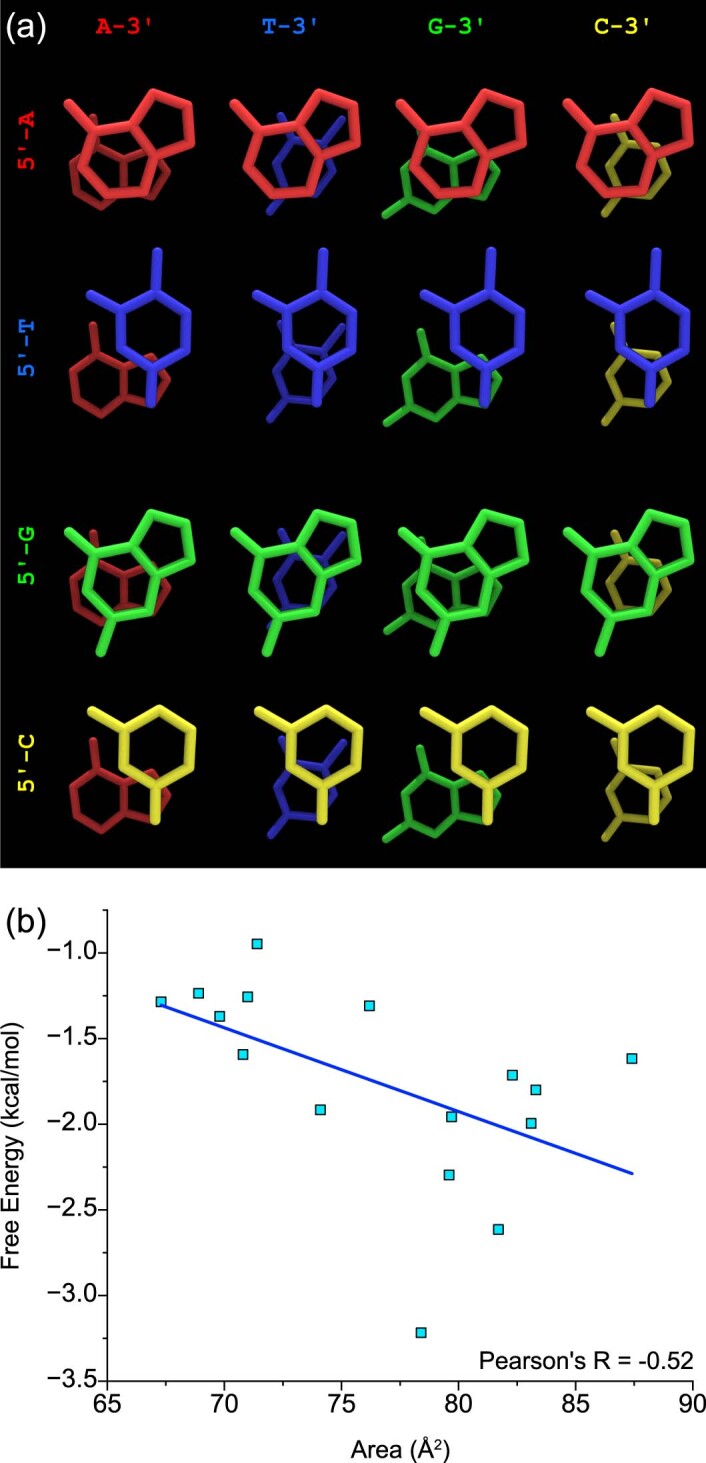
Correlation between molecular overlap and stacking energetics. **(a)** Top-view of nucleotide positioning from conventional B-form DNA for all 16 combinations. Foreground nucleotide is the nucleotide on the 5′ side. The background nucleotide is the nucleotide on the 3′ side. These combinations show clear distinction induced by directionality of the sequence. For example, A|C show larger overlap than C|A, in corroboration with around 250 and 10-fold off-rate enhancement, respectively. **(b)** Correlation plots between our measured values and the calculated overlap area between different dinucleotides involved in stacking in the B-form DNA (data from Pyshnyi and Ivanova Pyshnyi and Ivanova^2^). Source data

**Extended Data Fig. 6:**
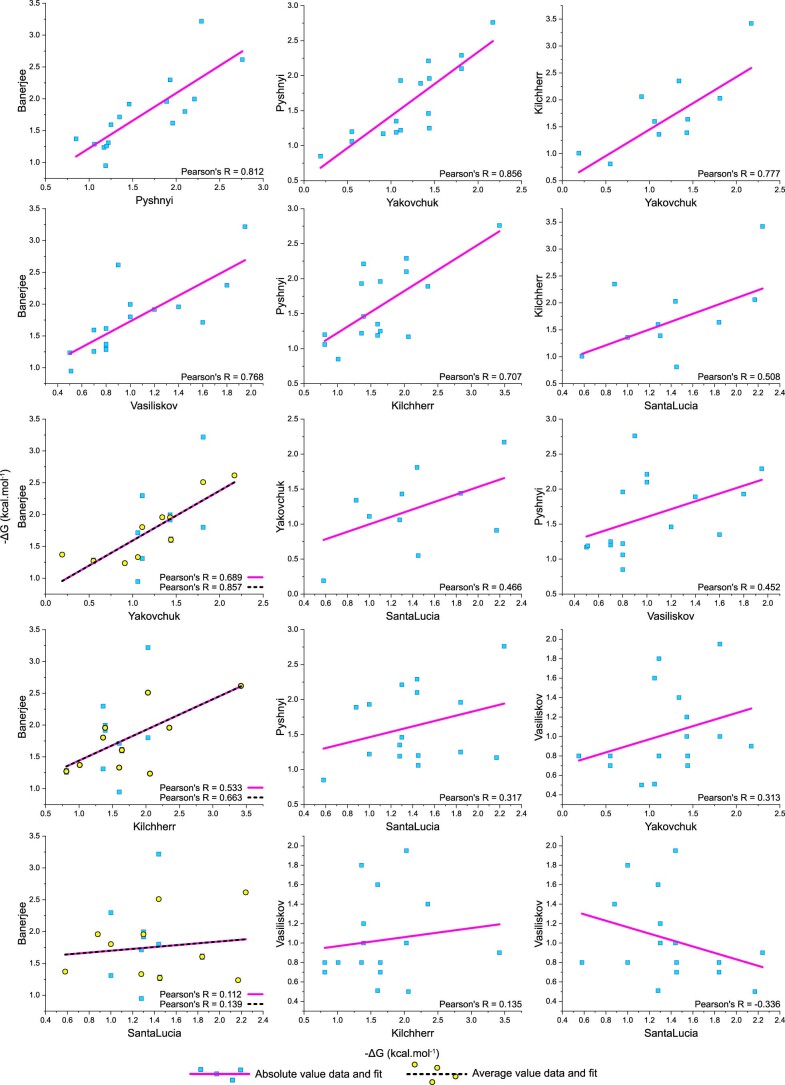
Correlation plots of different ΔG datasets outlined in Supplementary Table 3. Absolute values are shown in blue squares and the linear fit is plotted in magenta. The averaged values of our study have been shown in yellow circles and the liner fit is plotted as a dotted black line. Source data

By using the binding kinetics of the imager on stacking configuration and gap (Methods), we provide the absolute base-stacking free energy for each dinucleotide combination (Fig. 3b). The overall trend is that the base-stacking energetics presented here moderately correlate (Pearson’s *r* ≈ 0.52) to the degree of molecular overlap of nitrogenous aromatic rings in the dinucleotide combination (Extended Data Fig. 5). This fact is substantiated when comparing the swapped-sequence pairs that have the same molecular composition, such as A|C and C|A, G|C and C|G, but show distinct interactions because they interact via dissimilar exposed molecular surfaces (Extended Data Fig. 5). The reverse complement dinucleotides, such as A|C and G|T, C|T and A|G, also showed rather distinct stacking energies owing to the characteristic molecular interactions. This surprising observation was only possible as we could disentangle the individual dinucleotide base-stacking interactions rather than measuring complete base-pair stacking interactions^25,29^. Intriguingly, the average of our individually measured reverse complement sequence energetics is in close agreement with the previously reported base-pair stacking energetics (Extended Data Fig. 6 and Supplementary Table 3)^25,29,45,46^.

Our data are in qualitative agreement with previous results showing that purine|pyrimidine interactions are more stable than their counterparts^18^, but the absolute free energies are surprisingly higher^3,25,29,45^. This discrepancy is probably due to varied experimental strategies and conditions used across different studies. For example, our results were obtained at 21 °C, which considerably stabilizes the interactions compared with those obtained under 37 °C in earlier studies^3,36,46^. In accordance with previous findings^28^, we observe a trend in stacking energetics depending on the nucleotide at the 3′ end of the dinucleotide—highest for cytosine and lowest for guanine.

As the initiation of the imager strand dissociation requires breaking both the base-pairing and base-stacking interactions at the junction of imager and stem, we cannot completely rule out the cooperative effects of these two interactions leading to the observed energies^47^. We tested the cooperative effect by varying the sequence context and length of the imager or stem sequences (Extended Data Fig. 4). If cooperative effects were the determining factor of the measured energies, we expect our results to be affected by the sequence context. However, we observed very similar stacking energies independent of sequence, indicating that the underlined imager or stem sequences have a negligible effect (Extended Data Fig. 4). Taken together, these results substantiate that cooperative interactions minimally affect the measured base-stacking energies.

Although cross-stacking interactions between the nucleotides on the opposite strand of DNA partially contribute to base-stacking interactions^48^, the obtained energies are minimally affected by these interactions as they are extracted after comparing the gap and nick configurations. This is evident as the free energies calculated using the first exponent from the fitting of the stacking site binding events and the external gap are indistinguishable (Supplementary Fig. 8).

Interestingly, our single-molecule base-stacking energetics correlate well with previously published results (Fig. 3c and Extended Data Fig. 6)^3,26,29,36^. In contrast, the base-stacking energies from nearest-neighbour parameters^25,29^ that are used for calculating the stability of DNA duplexes poorly correlate with our and other published data (Fig. 3c). A likely explanation is that it is not possible to disentangle the base-pairing and base-stacking interactions from the nearest-neighbour parameters. More importantly, the current report presents individual base-stacking interactions, unlike the previous reports that estimated base-pair stacking interactions, which is a possible reason behind the observed differences^25,29,45,49^.

## Effects of chemical modifications on stacking energies

We measured the base-stacking energies between chemically modified nucleotides (Supplementary Table 4). While small chemical modifications, such as methylation, do not considerably affect the stacking interactions, modifications at the nucleotide level (for example, Cy3B fluorophore) result in a dramatic decrease in the overall energetics (Fig. 3d,e). This is probably due to the bulky nature of the fluorophore that could prevent the full degree of interactions between the two nitrogenous bases in a double-stranded DNA context. Such structural blemishes arising due to DNA damage could probably expose the DNA to further mutagenic degradation as it destabilizes the DNA by preventing proper base-stacking interactions.

We also tested the stacking interactions of a natural nucleoside, inosine, which is commonly found in transfer RNA and is known to form wobble base pairs with adenine and cytosine. We found that inosine stacking interactions are the weakest among all the measured dinucleotide pairs (Fig. 3f). These results demonstrate that our assay can be used to study a variety of nucleic acid modifications that play a crucial role in designing aptamers^50^ and other nucleosides.

## Stacking energies help assemble multimeric nanostructures

**Fig. 4:**
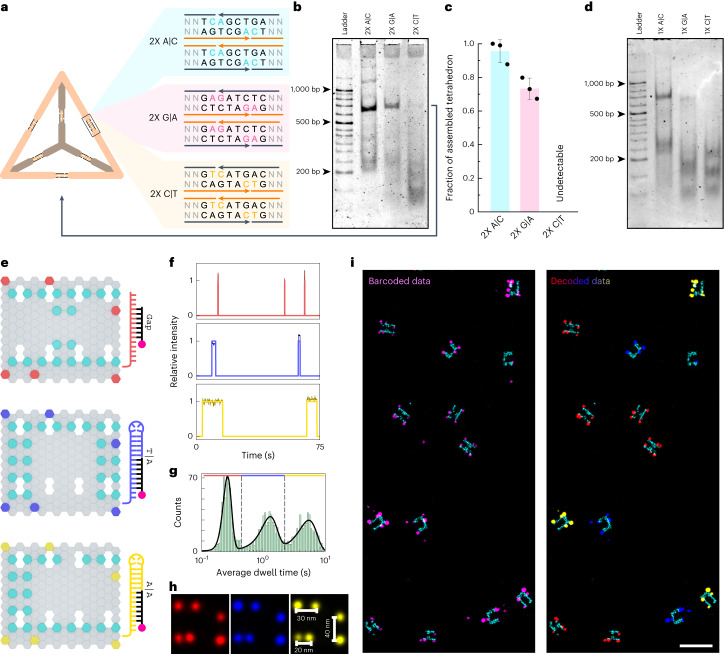
Application of base-stacking energetics for assembly of DNA nanostructures and multiplexed DNA-PAINT. **a**, Schematic design of tetrahedron structure and sequences carried by three arms. **b**, Polyacrylamide gel image showing tetrahedron structures. 2× A|C, 2× G|A and 2× C|T indicate the corresponding sequences involved as shown in **a**. **c**, Quantification of tetrahedron fraction compared with entire intensity in the given lane. Data represent mean and standard deviation of three individual datasets. **d**, Tetrahedron structures assembled from 1× stacking interactions. **e**, Schematics of origami structures for multiplexed DNA-PAINT imaging. Cyan extensions are for ground-truth identification. The design details of the coloured extensions are shown on the right side. Gap (red), T|A (blue) and A|A (yellow) are bound by the same imager with different binding strengths because of the different stacking or gap interactions, facilitating multiplexed imaging. **f**, Representative idealized imager binding time traces overlaid with relative photon count on each bound frame. **g**, Histogram showing average dwell-time distributions from all the origami structures in the field of view (*n* = 1,463). The dashed lines demarcate the peaks based on the expected average binding times and the coloured lines represent the gap or stack data. The colour code is same as in **e**. Darker shades represent the relative photon counts on each frame across each binding event. **h**, Overlaid DNA-PAINT origami structures taken from the demarcated histogram data. The colour code is similar to that stated in **e** (*n* = 525, *n* = 456 and *n* = 425 in the order of the images). **i**, Representative barcoded DNA-PAINT data (left) and decoded data (right) based on the average dwell times corresponding to the ground truth (cyan). The colour code is similar to that stated in **e**. Scale bar, 200 nm (**i**). Source data

**Extended Data Fig. 7:**
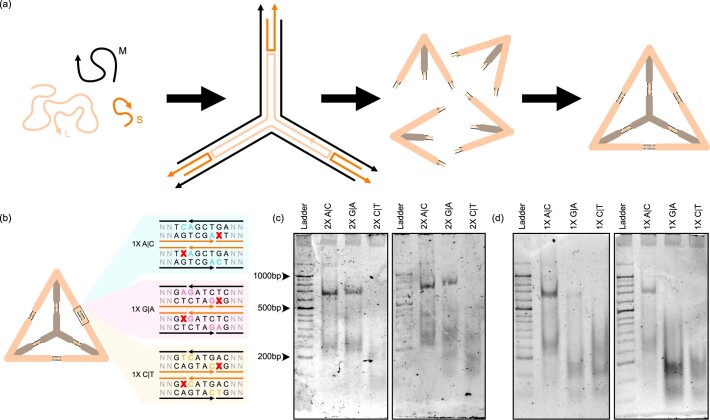
Tetrahedron folding strategy. **(a)** Each tetrahedron is made up of four subunits, each formed using three distinct DNA strands, namely L, M, and S. **(b)** Sequences at the interacting junctions for 1× A|C, 1× G|A and 1× C|T. **(c)** Other gel replicates showing folding efficiency of 2× A|C, 2× G|A, and 2× C|T. 2× A|C consistently shows greater fractions of assembled structures as compared to 2× G|A. Tetrahedrons formed with 2× C|T is undetectable. **(d)** Other gel replicates showing folding efficiency of 1× A|C, 1× G|A, and 1× C|T. 1× A|C consistently shows assembled structures. Tetrahedrons formed with 1× C|T and 1× G|A are undetectable. Source data

To investigate the role of stacking energies in assembling higher-order DNA nanostructures, we folded a DNA tetrahedron made up of three strands, each with four nucleotide sticky ends of the three arms (Extended Data Fig. 7a)^30,51^. We designed two sets of origami structures: one with two pairs of stacking interactions (2×) at each arm, and another with one pair of stacking and one gapped pair (1×) (Fig. 4a and Extended Data Fig. 7b). We tested A|C, G|A and C|T stackings in both cases, representing strongest, moderate and weakest stacking interactions.

As expected, we observed that the strongest stacking dinucleotide, A|C, efficiently stabilized tetrahedron structures with both 1× and 2× stackings at each arm (Fig. 4b–d and Extended Data Fig. 7b–d). While G|A stacking resulted in only observable tetrahedron structures with 2× stacking interactions, C|T interactions were not sufficient to stabilize detectable structures with 2× or 1× stacking dinucleotides. These results demonstrate the possibility of exploiting stacking energies for efficient design of higher-order DNA nanostructures. In addition, these data reconcile our measured energies where A|C, G|A and C|T represent strongest, moderate and weakest stacking energies.

## Stack-PAINT for multiplexed super-resolution imaging

To test the applicability of stacking interactions for simultaneous multiplexing in DNA-PAINT super-resolution imaging^52^, we envisioned ‘three colour’ multiplexing based on the tunable imager binding times (Supplementary Fig. 9a). For a proof-of-principle demonstration, we chose an imager strand with an adenine terminal nucleotide and three different docking strands in which one without a stem and two others form a stem with either adenine or thymine at the termini (that is, gap, A|A and T|A). On these configurations, the imager strands are expected to bind on average around 150 ms, 1.5 s and 8 s, respectively (Supplementary Fig. 9a). We designed three DNA-origami grids with docking-strand extensions including these stem configurations (Fig. 4e). The docking strands were separated by varying distances for testing the super-resolvability of multiplexed DNA-PAINT. As the binding kinetics are engineered based on the stacking nucleotides, we termed this modality Stack-PAINT. For ground-truth verification, we also decorated the origami structures with another set of docking strands in specific grid patterns (H, C and U grids; Fig. 4e).

Upon Stack-PAINT imaging, individual time traces of imager bindings showed the expected dwell times for each of the nick configuration (Fig. 4f). We obtained the average dwell times of the imager on individual origami structures in the entire field of view, the histogram of which showed three distinguishable populations as expected (Fig. 4g). We accordingly classified the origami structures under each peak into separate populations (Fig. 4g). The origami structures were then transformed into pseudo-coloured, barcoded images based on the matching dwell times. We ascertained that Stack-PAINT could resolve 20 nm separated docking strands using all three configurations (Fig. 4h). We matched the kinetically analysed Stack-PAINT data with the ground-truth grid patterns with a high decoding accuracy (~97%; Fig. 4i and Supplementary Fig. 9b–d), demonstrating the applicability of specific stacking interactions for multiplexed super-resolution imaging. Along similar lines, we envision that our stacking energetics data will enable the design of novel DNA-PAINT probes with tunable kinetics.

## Conclusions

This work presents direct measurement of a comprehensive list of dinucleotide base-stacking interactions, providing all 16 combinations. We developed a multiplexed, high-throughput single-molecule imaging assay exploiting the power of DNA nanotechnology to extract the binding kinetics of fluorophore-labelled short single-stranded DNA. As DNA-PAINT records the transient interactions of repetitive imager binding on the docking strand under equilibrium, we obtained about half a million molecular hybridization events, which is required for robust statistical analysis of binding kinetics^53^, thereby facilitating the deduction of the absolute dinucleotide base-stacking energetics with high accuracy of ±0.1 kcal mol^−1^. We carefully analysed the kinetics under orthogonal configurations to ascertain our measured values with high confidence.

Recently, a parallel study used a single-molecule force-spectroscopic assay to measure individual base-stacking energetics of ten different dinucleotide combinations^30,31^. However, a few stacking energies in these non-equilibrium measurements deviate from our results, the origins of which are unknown. We have observed that A|C stacking is more efficient in constructing multimeric DNA nanostructures compared with G|A stacking, which was reported as the strongest by the force spectroscopy study^31^. This outcome fortifies the reliability of our data. Furthermore, we also account for the directionality of the stacking interactions, which according to our data, plays a crucial role in determining the stacking energy. The measured individual dinucleotide stacking free energies are relevant in the canonical DNA context but may not apply to free nucleotide interactions.

Given that the base-stacking interactions provide great control over the imager binding times, we exploited this engineered kinetics for the designing of DNA-PAINT imaging probes for multiplexed high-resolution microscopy. In combination with the sequence of the imager and salinity of the buffer, base-stacking energetics provide multifaceted control over the binding kinetics of the imager strands and will drive the development of novel DNA-PAINT probes.

Previous reports focusing on stitching DNA nanostructures utilized base-stacking energies without prior knowledge^6,13–15^. In this study, we exploited the known base-stacking energies for efficient oligomerization of DNA nanostructures, demonstrating that these precise stacking energies can facilitate the design and creation of multi-subunit DNA nanostructures with enhanced precision. As the multiplexed imaging strategy requires a widely used TIRF microscope, it can readily be extended to study other nucleic acid interactions that might contain different chemically modifications.

## Methods

### Slide preparation

Microscopy slide and coverslip preparation was done as previously described^39,43^. The slides (VWR 631-1550) were drilled using a diamond head drill bit (Meisinger 801-009-HP) and drill gun (DigitalCraft LRUXOR). In case the slides were being reused, macroscopic particles were removed using 5% v/v dish washing detergent before further steps were performed. Slides and coverslips (VWR 631-0147) were initially rinsed thoroughly using MilliQ water and then immersed into coplin jars (Tarsons 480000) containing MilliQ water. The slides and coverslips were then sonicated for 5 min (IGene LabServe IGNUC-9). MilliQ water was then replaced with acetone (SRL 31566) twice and was sonicated for 5 min. Acetone was then replaced with MilliQ water followed by 1 M KOH (BDH 296228) and sonicated for at least 30 min. The slides and coverslips were then rinsed using MilliQ water by replacing the KOH in the coplin jars at least three times. The slides and coverslips were then sonicated in MilliQ water for 10 min and dried using compressed nitrogen gas.

Piranha etching was performed on the slides and coverslips. Piranha solution was prepared by adding one part of 30% H_2_O_2_ (EMPLURA 107209) to three parts of H_2_SO_4_ (Fisher Scientific 29997). This solution was then transferred into the coplin jars and left for 30 min. The piranha solution was disposed of in a dedicated waste container, and the slides and coverslips were rinsed thoroughly using MilliQ water, then rinsed with methanol twice and sonicated in methanol for 20 min.

After this, aminosilanation was performed. The aminosilanation mix was prepared by mixing 5 ml acetic acid (SDFCL 20001) in 100 ml methanol, followed by addition of 10 ml (3-aminopropyl) triethoxysilane (SRL 33993 or TCI A0439) and mixed thoroughly. This solution was then poured over the slides and coverslips held within the coplin jar and left for 25 min. The slides and coverslips were washed thoroughly with fresh methanol three times followed by rinsing with MilliQ water. Slides were then dried with compressed nitrogen gas.

Passivation was performed on these aminosilanated slides using mPEG-SVA (succinimidyl valerate) (Laysan Bio mPEG-SVA-5000) and biotin-PEG-SVA (Laysan Bio Biotin-PEG-SVA-5000) at a 40:1 mass ratio in 0.1 M NaHCO_3_ (Sigma S5761) pH 8.5. For 15 pairs of slides and coverslips, 120 mg mPEG-SVA and 3 mg biotin-PEG-SVA were dissolved in 960 µl of 0.1 M NaHCO_3_. Then 60 µl of this solution was added onto each slide and then sandwiched by placing a coverslip over it gently. These slides and coverslips were stored in humid chambers for 8–12 h at room temperature (21 °C) under dark. The following day, the sandwich was disassembled and washed thoroughly with MilliQ water. These slides were then dried with compressed nitrogen gas and stored inside 50 ml centrifuge tubes (NUNC 339653) and stored under inert conditions in nitrogen gas.

The flow cells were assembled using double-sided tape (3 M Scotch 136D MDEU) on the PEG-passivated surface of the sides. Coverslips were placed on the taped slide ensuring that the PEG-passivated surfaces of the slide and coverslip make up the interiors of the microfluidic flow cells. The open edges of these channels were sealed using epoxy (Araldite Klear).

### DNA-origami folding

We used the Picasso design module^33^ to get the staple sequences of specific extensions for the grids and blank staples (Supplementary Table 8, sheets 1 and 2) for preparing rectangular DNA-origami nanostructures. M13mp18 single-stranded DNA (Bayou Biolabs P107) was used as the scaffold for the DNA origami. Biotinylated oligonucleotide staples (Supplementary Table 8, sheet 3) were used to anchor the origami structure to the flow cell surface. Staples with extension for the gap and nick positions were designed using sequences from Picasso design and CadNanoSQ (https://cadnano.org/; Supplementary Table 8, sheets 4 and 5). Folding buffer contained 50 mM Tris-Cl (Tris-Base, Sigma 77861; HCl, Fisher Scientific 29507) pH 8.0, 12.5 mM MgCl_2_ (EMPLURA 105833) and 0.2 mM EDTA (SRL 35888). We set up 30 µl reactions with 10 nM scaffold DNA, 100 nM biotin staples, 100 nM blank staples, 1 µM staples for the grid and 33.3 µM gap- or nick-specific staple in the folding buffer. The mix was then heated up to 80 °C, held for 5 min, and then cooled to 4 °C, in steps of 0.1 °C every 5 s in a thermocycler.

The folded origami structures were purified using Sartorius Vivaspin 500 (Sartorius VS0132) centrifugal filters. Equilibration was performed by spinning the columns at 3,000*g* for 5 min with 500 µl HPLC-grade water (SRL 92605) followed by 500 µl folding buffer. The resultant origami mix was then applied to the column along with 500 µl folding buffer at 800–1,000*g* for three rounds, to remove most of the unincorporated staples. Origami structures were stored in the folding buffer at a concentration of 3.3 nM at −20 °C.

### Imager fluorophore conjugation and purification

We obtained 3′-end amine modified oligonucleotides from Sigma (Supplementary Table 8, sheet 6) and dissolved to a final concentration of 1 mM using HPLC-grade water.

Fluorophores were dissolved in DMSO (Sigma D8418) to the following concentrations. Cy3B-MonoNHS-Ester (Cytiva PA63101) was dissolved to a final concentration of 13 mM, Atto647N-MonoNHS-Ester (Sigma 18373-1MG-F) was dissolved to a final concentration of 11.8 mM and Cy5-MonoNHS-Ester (Cytiva PA15101) was dissolved to a final concentration of 1.3 mM.

A 10× PBS was prepared by adding 80 g NaCl (SRL 3205), 2 g KCl (Sigma P9541-1KG), 14.4 g Na_2_HPO_4_ (SRL 1949146) and 2.4 g KH_2_PO_4_ (Ranbaxy 5HEV0740) in 1 l of MilliQ water followed by autoclaving at 121 °C for 15 min. The pH of the buffer was adjusted to 7.4.

For conjugation of Cy3B or Atto647N to the imager, 15 nM DNA (15 µl from 1 mM stock) was dissolved in a mixture containing 3 µl 10× PBS, 3 µl of 1 M NaHCO_3_ and 3.24 µl HPLC-grade water. Seventy-five nanomoles (5.76 µl of 13 mM Cy3B or 6.35 µl of 11.8 mM Atto647N) of fluorophore was added to the above mixture and vortexed vigorously. The mix was incubated overnight in the dark at room temperature under vigorous shaking.

For conjugation of Cy5 to the imager, 2 nM DNA (2 µl from 1 mM stock) was dissolved in a mixture containing 1.5 µl 10× PBS, 1.5 µl of 1 M NaHCO_3_ and 4 µl HPLC-grade water. Then 7.8 nM (6 µl of 1.3 mM Cy5) fluorophore was added to the above mixture and vortexed vigorously. The mix was incubated overnight in the dark at room temperature under vigorous shaking.

Post overnight incubation, the conjugated product was purified from the free fluorophore and unconjugated DNA oligonucleotide using reverse-phase (Phenomenex 00B-4442-E0 Clarity 5 µm Oligo-RP, LC column 50 × 4.6 mm, Ea) HPLC (Agilent Technologies). The conjugated product was then dissolved in HPLC-grade water and stored at −20 °C.

### Sample preparation

Microfluidic flow cells with a PEG-passivated coverslip and slide were incubated with 10 µl of 0.2 mg ml^−1^ neutravidin (Sigma 31000) in T50 buffer containing 50 mM Tris-Cl pH 8.0, 50 mM NaCl and 0.2 mM EDTA for 20 min. This was followed by thorough washing of the microfluidic channel with 600 µl of T50 buffer. Imaging/immobilization buffer (buffer I) containing 50 mM Tri-Cl pH 8.0, 10 mM MgCl_2_ and 0.2 mM EDTA was used to wash the channel before origami immobilization. The origamis intended for a specific imaging run were pooled together at a final concentration of 400–600 pM each in buffer I and applied onto the channel and incubated for 20 min. This was followed by washing of the channel with 600 µl buffer I before imaging to remove any unbound origamis.

### Microscopy and imaging

Microscopy was performed on a Nikon Ti2 Eclipse microscope equipped with a motorized H-TIRF, perfect focus system and a Teledyne Photometrics PRIME BSI sCMOS camera. Illumination using 561 nm and 640 nm wavelength lasers was done using the L6cc laser combiner from Oxxius. Imaging was done under total internal reflection conditions. An oil immersion objective lens (Nikon Instruments Apo SR TIRF 100×, numerical aperture 1.49, oil) was used for imaging. Imaging was performed with 2 × 2 binning of pixels and the camera was cropped to an effective size of 512 × 512 pixels, each pixel spanning 130 × 130 nm. Acquisition was done by setting the camera to a readout sensitivity of 16 bit. Imaging parameters used in the different experiments are outlined in Supplementary Table 7.

A solution of 20× PCD was made by dissolving PCD (protocatechuate 3,4-dioxygenase; Sigma P8279-25UN) in buffer containing stock in 50 mM KCl, 1 mM EDTA and 100 mM Tris–HCl, pH 8.0 and 50% glycerol (Sigma G5516-1L) to a final concentration of 6 µM. The solution was divided into 10 µl aliquots in PCR tubes and stored at −20 °C.

A solution of 40× PCA was made by dissolving 154 mg of PCA (protocatechuic acid/3,4-dihydroxybenzoic acid; Sigma 37580-100G-F) in 10 ml HPLC-grade water adjusted to pH 9.0 using 1 M NaOH (SRL 96311). The solution was divided into 100 µl aliquots in 200 µl tubes and stored at −20 °C.

A solution of 100× trolox was prepared by dissolving 100 mg of trolox (6-hydroxy-2,5,7,8-tetramethylchroman-2-carboxylic acid; Sigma 238813-1 G) in 430 µl methanol, 345 µl of 1 M NaOH and 3.2 ml HPLC-grade water. The solution was divided into 20 µl aliquots in PCR tubes and stored at −20 °C.

Before imaging, 100 µl of imaging buffer was prepared in buffer I with a final concentration of 1× PCA, 1× PCD and 1× trolox, along with imagers at concentrations mentioned in Supplementary Table 7. Imaging was always performed with the 640 nm laser before the 561 nm laser to minimize the effect of Atto647N or Cy5 fluorophore photobleaching by the higher-energy (561 nm) light source.

### Imaging of stem-loop layout

Origamis were folded in the same manner as mentioned above (Supplementary Table 8, sheets 1 and 7–9). Imaging was performed using Exchange-PAINT^54^, where the first round of imaging was done to image the origamis carrying R1×5 extensions followed by origamis carrying R4×5 extensions with appropriate imagers carrying Atto647N fluorophore at the mentioned concentrations (Supplementary Table 7). Imaging buffer was prepared as mentioned above. Subsequent imaging rounds were separated by washing with 2 ml imaging buffer. Finally, the nick and gap were measured using Cy3B-labelled imager at the concentrations mentioned in the Supplementary Table 7.

### Data analysis

The obtained raw fluorescence data were reconstructed using the Picasso Localize software package^33^ to obtain a super-resolved image. Drift correction in *X*–*Y* was performed by redundant cross-correlation. Redundant cross-correlation was also used to align the super-resolved structures from the two imaging channels (Supplementary Fig. 1). Origamis were manually picked based on their grid structures. Localizations from the gap or nick spots from the assay sites were extracted using Picasso Render for further kinetics analysis. We performed kinetic analysis using a custom-written MATLAB code. Briefly, the list of localizations exported for each origami pick was further analysed for individual dwell times and dark times. Dwell times were calculated based on the presence of consecutive binding events with a gap of not more than 25 frames (that is, 1,250 ms; Fig. 1f). This is done to overcome any potential flickering of the fluorophore and the lower signal-to-noise ratios that arise due to the lower laser powers that were used during imaging to maintain the photostability of the fluorophore (Supplementary Fig. 8). Further, this will unlikely combine two consecutive binding events due to the long time intervals of around 100 s to 200 s between consecutive bindings (Supplementary Fig. 9). Bootstrapping for 100 iterations was performed based on the mean using MATLAB’s built-in bootstrap function on the list of individual dwell times for each dataset separately.

Dark times were calculated based on the durations between two consecutive binding events (Fig. 1f). The obtained dark times were bootstrapped with parameters mentioned above and used for further kinetic analysis.

### Kinetic analysis for calculating the base-stacking energies

Here we describe the kinetic analysis of the single-molecule imaging data. The set of bootstrapped dwell times were plotted on histogram and then fit with a mono-exponential curve $(y={a}_{1}\times {{\mathrm{e}}}^{{k}_{{{\mathrm{off}}}}{t}})$ in case of the gap, resulting in the off-rate constant *k*_off,gap_. For the nick configuration data, we fit them with a bi-exponential curve $(y={a}_{1}\times {{\mathrm{e}}}^{{k}_{{{{\mathrm{off}}}}{,1}}{t}}+{a}_{2}\times {{\mathrm{e}}}^{{k}_{{{{\mathrm{off}}}}{,2}}{t}})$ using gap off-rate as proxy for fitting the first exponential. The distribution of individual dwell times shown in Supplementary Fig. 4a clearly shows that the imager binds on the docking strand in two modes—one without the terminal nucleotide stacking on the stem, leading to a faster decaying population, and the second with terminal nucleotides stacking, leading to a slower decaying population away from the first population (Supplementary Fig. 4a). As we observed two distinct populations in the individual dwell-time distributions, we assumed that the unstacked binding mode was due to the stem undergoing fraying temporarily; the mechanistic details behind this are still unclear, although similar observations have been reported by other studies^41,42^. The fraying of the stem is mechanistically equivalent of the gap construct. This fitting results in two off-rate constants (*k*_off,1_ and *k*_off,2_) in which *k*_off,1_ denotes the dissociation from unstacked-bound state to the unbound state, which closely resembles the gap off-rate constant. The second off-rate constant, *k*_off,2_, depicts the imager’s apparent dissociation rate from the bound state, which comprises of bound-stacked and bound-unstacked states to the unbound state (Supplementary Fig. 5). This kinetic analysis provides us with the off-rate constants of unstacked- and stacked-bound states based on the experimentally measured dwell times.

In a similar manner, we also obtained the binding rate constant (*k*_bind_) by kinetic analysis of the dark-time distributions. We built a histogram of the bootstrapped dark times and fit with a mono-exponential curve $(y={b}_{1}\times {{\mathrm{e}}}^{{k}_{{{\mathrm{bind}}}}{t}})$ to obtain the individual binding rate constants for the gap and the nick configurations.

The gap configuration is modelled with a bound state and an unbound state with corresponding binding rate (*k*_bind,gap_) and dissociation rate (*k*_off_). On the nick configuration, imager binding is represented by rate constant *k*_bind_ in the ‘frayed’ stem state or the intact state. The frayed state resembles the gap configuration. When the stem is intact, the imager binds in either the stacked or the unstacked state with rate constants *k*_on,st_ and *k*_on,unst_, respectively. Once in the bound state, the imager may undergo stacked-to-unstacked transitions and vice versa as indicated within the binding event; while doing so, the imager may dissociate from either the stacked or the unstacked configuration with rate constants represented by *k*_off,st_ and *k*_off,unst_, respectively (Supplementary Fig. 5). We have neglected the stacked-unpaired and the unstacked-unpaired states (both of which trigger dissociation) whose occupancies would be relatively low.

The apparent rate of dissociation (*k*_off,2_) from the bound state can be written as

$${k}_{{{\mathrm{off}}},2}=\left(\frac{{N}_{{{\mathrm{st}}}}}{{N}_{{{\mathrm{st}}}}+{N}_{{{\mathrm{unst}}}}}\right){k}_{{{\mathrm{off}}},{{\mathrm{st}}}}+\left(\frac{{N}_{{{\mathrm{unst}}}}}{{N}_{{{\mathrm{st}}}}+{N}_{{{\mathrm{unst}}}}}\right){k}_{{{\mathrm{off}}},{{\mathrm{unst}}}}$$

where *N*_st_ and *N*_unst_ are occupancies of stacked and unstacked states during the bound state.

*k*_off,st_ is rate of dissociation from the stacked state and *k*_off,unst_ is the rate of dissociation from the unstacked state.

$${k}_{{{\mathrm{off}}},2}\left({N}_{{{\mathrm{st}}}}+{N}_{{{\mathrm{unst}}}}\right)={N}_{{{\mathrm{st}}}}{k}_{{{\mathrm{off}}},{{\mathrm{st}}}}+{N}_{{{\mathrm{unst}}}}{k}_{{{\mathrm{off}}},{{\mathrm{unst}}}}$$

$$\frac{\left({N}_{{{\mathrm{st}}}}+{N}_{{{\mathrm{unst}}}}\right)}{{N}_{{{\mathrm{unst}}}}}=\frac{{N}_{{{\mathrm{st}}}}{k}_{{{\mathrm{off}}},{{\mathrm{st}}}}+{N}_{{{\mathrm{unst}}}}{k}_{{{\mathrm{off}}},{{\mathrm{unst}}}}}{{N}_{{{\mathrm{unst}}}}\,{k}_{{{\mathrm{off}}},2}}$$

$$\frac{{N}_{{{\mathrm{st}}}}}{{N}_{{{\mathrm{unst}}}}}+1=\frac{{N}_{{{\mathrm{st}}}}}{{N}_{{{\mathrm{unst}}}}}\frac{{k}_{{{\mathrm{off}}},{{\mathrm{st}}}}}{{k}_{{{\mathrm{off}}},2}}+\frac{{k}_{{{\mathrm{off}}},{{\mathrm{unst}}}}}{{k}_{{{\mathrm{off}}},2}}$$

$$\frac{{N}_{{{\mathrm{st}}}}}{{N}_{{{\mathrm{unst}}}}}\left(1-\frac{{k}_{{{\mathrm{off}}},{{\mathrm{st}}}}}{{k}_{{{\mathrm{off}}},2}}\right)=\frac{{k}_{{{\mathrm{off}}},{{\mathrm{unst}}}}}{{k}_{{{\mathrm{off}}},2}}-1$$

$$\frac{{N}_{{{\mathrm{st}}}}}{{N}_{{{\mathrm{unst}}}}}=\frac{\left(\frac{{k}_{{{\mathrm{off}}},{{\mathrm{unst}}}}}{{k}_{{{\mathrm{off}}},2}}-1\right)}{\left(1-\frac{{k}_{{{\mathrm{off}}},{{\mathrm{st}}}}}{{k}_{{{\mathrm{off}}},2}}\right)}$$

$$\frac{{N}_{{{\mathrm{st}}}}}{{N}_{{{\mathrm{unst}}}}}=\frac{{k}_{{{\mathrm{off}}},{{\mathrm{unst}}}}-{k}_{{{\mathrm{off}}},2}}{{k}_{{{\mathrm{off}}},2}-{k}_{{{\mathrm{off}}},{{\mathrm{st}}}}}$$

Assuming that *k*_off,st_ is very slow compared with *k*_off,2_, we discard *k*_off,st_ in the denominator. That provides us with the following equation.

$$\frac{{N}_{{{\mathrm{st}}}}}{{N}_{{{\mathrm{unst}}}}}=\frac{{k}_{{{\mathrm{off}}},{{\mathrm{unst}}}}-{k}_{{{\mathrm{off}}},2}}{{k}_{{{\mathrm{off}}},2}}$$

We are assuming that *k*_off,unst_ and *k*_off,gap_ are equivalent, which is a reasonable assumption because the imager in both configurations carry the same base pairs without terminal nucleotides stacking and we experimentally obtained *k*_off,gap_. We also have obtained *k*_off,2_ from experiments, which is the dissociation rate from the bound state.

Hence

1 $$\frac{{N}_{{{\mathrm{st}}}}}{{N}_{{{\mathrm{unst}}}}}=\frac{{k}_{{{\mathrm{off}}},{{\mathrm{gap}}}}-{k}_{{{\mathrm{off}}},2}}{{k}_{{{\mathrm{off}}},2}}$$

This ratio of stacked-to-unstacked occupancies in the bound configuration can be converted to the free energy of base stacking by taking Boltzmann’s weightage over it.

Therefore, the free energy of dinucleotide base stacking is given by the following equation.

2 $$\Delta {G}_{{{\mathrm{stack}}}}=-{kT}\,{\rm{ln}}\left(\frac{{N}_{{{\mathrm{st}}}}}{{N}_{{{\mathrm{unst}}}}}\right)$$

where *k* is the Boltzmann constant and *T* is the absolute temperature.

Plugging equation (1) in to equation (2) provides us the free energy of base stacking.

3 $$\Delta {G}_{{{\mathrm{stack}}}}=-{kT}\,{\rm{ln}}\left(\frac{{k}_{{{\mathrm{off}}},{{\mathrm{gap}}}}-{k}_{{{\mathrm{off}}},2}}{{k}_{{{\mathrm{off}}},2}}\right)$$

We calculated all the dinucleotide stacking free energies using the above equation. We note that a similar formalism was applied by ref. ^3^ for extracting the stacking free energies after gel electrophoresing of gapped, nicked and intact DNA molecules.

Note that the Δ*G*_stack_ is independent of the imager concentration as it only depends on the off-rate constants. The absolute temperature *T* is constant. If the temperature, concentration and salinity of the buffer in independent experiments are not well controlled, larger variations are expected in the free-energy estimations as the binding dynamics of short oligonucleotides are strongly dependent on these parameters^40^. The multiplexed experiments utilized in the current study are resistant to such possible variations as the normalization is internal to individual imaging experiments.

### Photobleaching calculation

Origamis carrying the S1 docking sequence (Supplementary Table 8, sheet 10) were folded using methods mentioned above. DNA origamis were immobilized on flow cells treated with neutravidin as mentioned above. Complimentary S1 strand carrying Cy3B was flown in at 1 pM concentration in buffer I. After 10 min of incubation, the flow cells were washed with 1 ml of buffer I to remove any unbound complimentary S1 strands carrying Cy3B. Imaging buffer was prepared with 1× PCA, 1× PCD and 1× trolox. For recreating the exact same conditions of the Cy3B imaging round (second imaging round), the imaging buffer was added to the flow channel and a dummy imaging run was performed for the duration of the first imaging rounds. This was followed by imaging of the stably bound Cy3B with 561 nm laser excitation in TIRF mode at a similar power to the experiments depicted in Fig. 3 and mentioned in Supplementary Table 7.

The acquired image was run through the Picasso Localize^33^ package to track individual fluorophores over a time course. Individual photobleaching times were obtained from the time traces generated similar to image analysis described above. These individual bleaching times were then plotted in a histogram. The fluorophores surviving throughout the imaging run and a small population of fluorophores that bleach during the first 100 s (first bin in Supplementary Fig. 8) were ignored for exponential fitting. The plotted events were then fit with a mono-exponential curve ($y={a\times }{{\mathrm{e}}}^{{k}_{{{\mathrm{photobleach}}}}{t}}$) to obtain the photobleaching rate.

### Tetrahedron folding and analysis

The tetrahedron origami structures are composed of three different DNA strands, namely L, M and S^30,51^. These strands were designed to carry different stacking ends (Fig. 4a). The sequences of strands L, M and S are shown in Supplementary Table 5. These strands were pooled in the order shown in Supplementary Table 6 in a 1:3:3 ratio. The pooled mixtures were heated to 95 °C and let to cool over a period of 48 h in an insulated water tub in 1× TAE/Mg^2+^ buffer composed of 40 mM Tris, pH 8.0, 20 mM acetic acid, 2 mM EDTA and 12.5 mM magnesium acetate. The structures were then stored at −20 °C. The samples were thawed at room temperature 20 min before loading on the gel. A 1-mm-thick 4% polyacrylamide gel (29:1) prepared in 1× TAE/Mg^2+^ buffer was used for analysing structure formation. Then 20 µl of sample along with 5 µl loading dye containing 0.003% bromophenol blue and 60% glycerol in 1× TAE/Mg^2+^ buffer was loaded in each well. For size reference, a 50 bp plus DNA ladder (dxbidt R4006) that was diluted by 20 times in 1× TAE/Mg^2+^ buffer before loading was used. The gels were run at constant voltage for 120 min at 4 °C in a cold room. The gels were then stained using GelRed (Biotium 41003) stain diluted to 3× in 0.1 M NaCl solution for 30 min on a gel rocker. The gels were then visualized in an ultraviolet transilluminator and quantified using BioRad Image Labs 6.1.

Quantification of the fraction of assembled tetrahedron was performed by dividing the tetrahedron band intensity by the total intensity of the same lane after background subtraction. The obtained values were then normalized with the greatest obtained fraction to quantify the fraction of assembled tetrahedron in each experimental triplicate.

### Stack-PAINT imaging

Origami structures were folded with staples defined in Supplementary Table 8, sheet 11, and purified using centrifugal filtration. The origami samples were immobilized on the surface of a PEG-passivated glass slide as described above. Imaging was performed as mentioned in Supplementary Table 7. Reconstructed data were aligned for both channels. Origamis were picked based on the six extensions that were placed for resolution testing and then filtered based on the presence of the underlying grid. The mean dwell times at each picked origami structure was calculated. We then constructed a histogram from the mean dwell times and fitted with a triple Gaussian curve using the MATLAB ‘gauss3’ function. Each of the three Gaussian peaks was split into three datasets by manual demarcation based on the known dwell-time averages over each pick. For assessing the robustness of our decoding technique, we manually filtered the selected origami structures based on the grid structures. We then compared the manual selection set with the set delineated based on the average dwell times. We calculated the error in calling the correct origami by taking the ratio of origami numbers that fall outside the expected region to total origami structures analysed.

### Statistics and reproducibility

No statistical method was used to predetermine the sample size. Each stacking experiment was performed in triplicate. As evident from the data, the results were highly reproducible.

## Online content

Any methods, additional references, Nature Portfolio reporting summaries, source data, extended data, supplementary information, acknowledgements, peer review information; details of author contributions and competing interests; and statements of data and code availability are available at 10.1038/s41565-023-01485-1.

### Supplementary information

Supplementary Information Supplementary Figs. 1–9 and Tables 1–7.

Supplementary Table 8 This excel file contains the scaffold strand sequence and staple strand sequences used in the study.

### Source data

Source Data Fig. 1 Source data.

Source Data Fig. 2 Source data.

Source Data Fig. 3 Source data.

Source Data Fig. 4 Source data.

Source Data Extended Data Fig. 2 Source data.

Source Data Extended Data Fig. 4 Source data.

Source Data Extended Data Fig. 5 Source data.

Source Data Extended Data Fig. 6 Source data.

Source Data Extended Data Fig. 7 Uncropped gel images.

## Data Availability

All DNA-PAINT raw data are available on reasonable request. All the localization data obtained from analysis of raw DNA-PAINT data are deposited at https://zenodo.org/record/8090944. Kinetic rate constants and free energy of base-stacking interactions are included in Supplementary Information. Source data are provided with this paper.

## Code availability

DNA-PAINT raw data were analysed and visualized using the Picasso software (https://github.com/jungmannlab/picasso). The kinetic analysis code written in MATLAB (MathWorks) is available upon request from the authors.
