# Supplementary material for: Single-molecule analysis of DNA base-stacking energetics using patterned DNA nanostructures
Source: Nat Nanotechnol. 2023 Aug 17;18(12):1474–82. doi: 10.1038/s41565-023-01485-1 (PMC10716042; doi:10.1038/s41565-023-01485-1)
Supplement: Supplementary file 1 — Supplementary Figs. 1–9 and Tables 1–7. [file 41565_2023_1485_MOESM1_ESM.pdf]

nature nanotechnology

Article https://doi.org/10.1038/s41565-023-01485-1

Single-molecule analysis of DNA base-stacking energetics using patterned DNA nanostructures

In the format provided by the authors and unedited

S. No.	Content
1	Overview DNA-PAINT images and traces of Gap and Nick
2	Overview and individual origamis showing five different barcoded shapes
3	Exponential fits to obtain dwell times for other replicates as shown in Fig 3a
4	Exponential fits to obtain dwell times for data shown in Extended Data Fig 4
5	Photobleaching characterization of Cy3B under the used imaging conditions
6	Exponential fits to dark times of all the stackings in all three replicates
7	Correlation plots comparing on rate enhancements to stacking energies and off rate
8	Comparison of free energy calculations from internal and external gaps
9	Stack-PAINT
10	Table outlining all the off rates obtained from the experiments
11	Table outlining all the binding rates obtained from the experiments
12	Tabulated base stacking values comparing published measurements
13	Table outlining all the off rates obtained from the experiments ploted in Fig 3d-f
14	Sequences of oligonucleotides used in multimeric tetrahedron assembly
15	Folding scheme of the tetrahedrons DNA origami structures
16	Imaging Parameters
17	Supplementary References

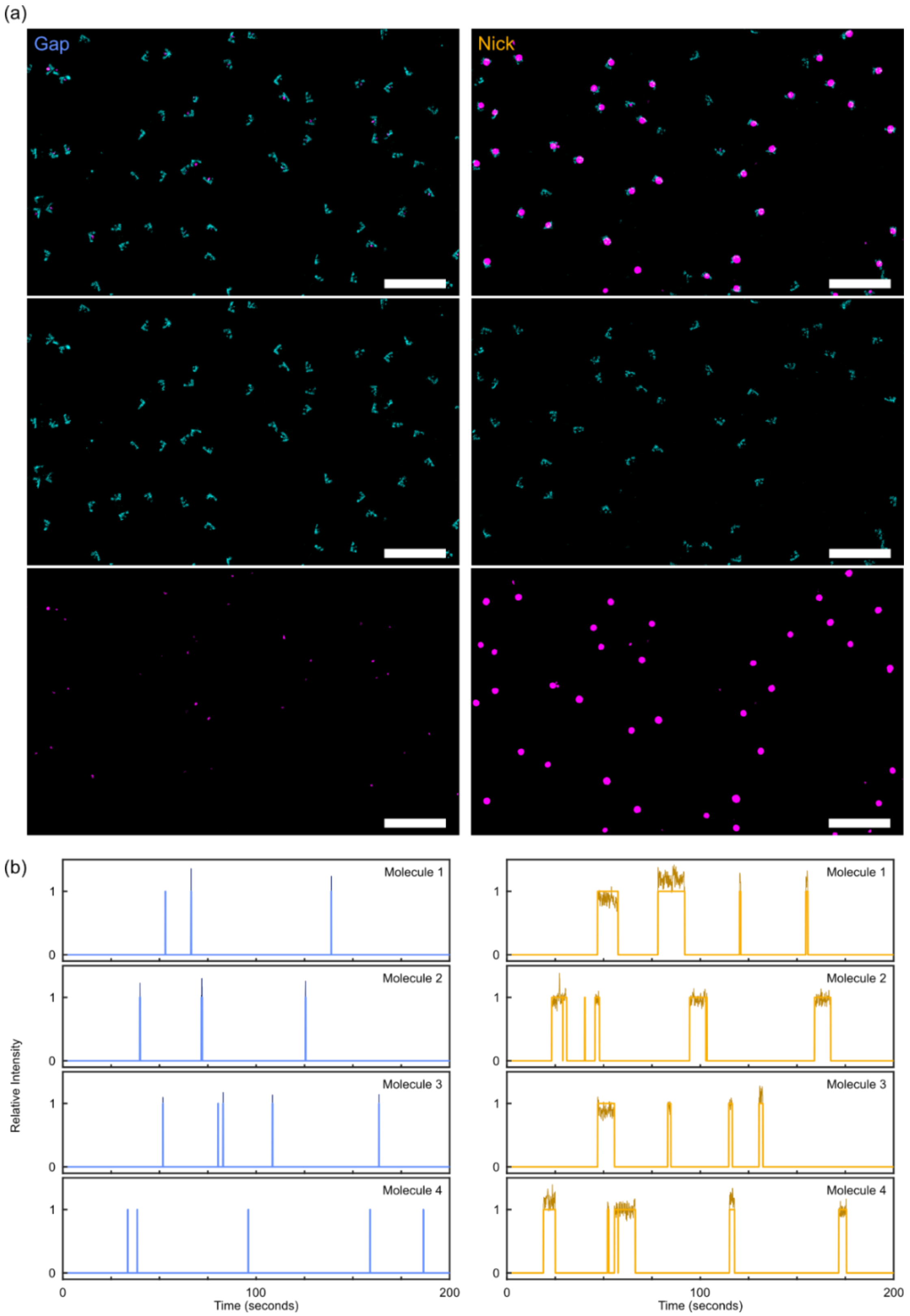

Supplementary Figure 1: Overview DNA-PAINT images showing the imager strand binding at the assay site (a) Large field of view showing grid and assay site under gap (left) and nick (right). Combined image (top) was obtained by overlaying the DNA-PAINT grid image (middle) with the assay site image (bottom). (b) Binding time traces for four example assay sites showing short bindings in the case of gap (left) and long bindings in the case of nick (right). Darker shades represent the relative photon counts in each frame across each binding event. (Scale Bar: 500 nm (a))

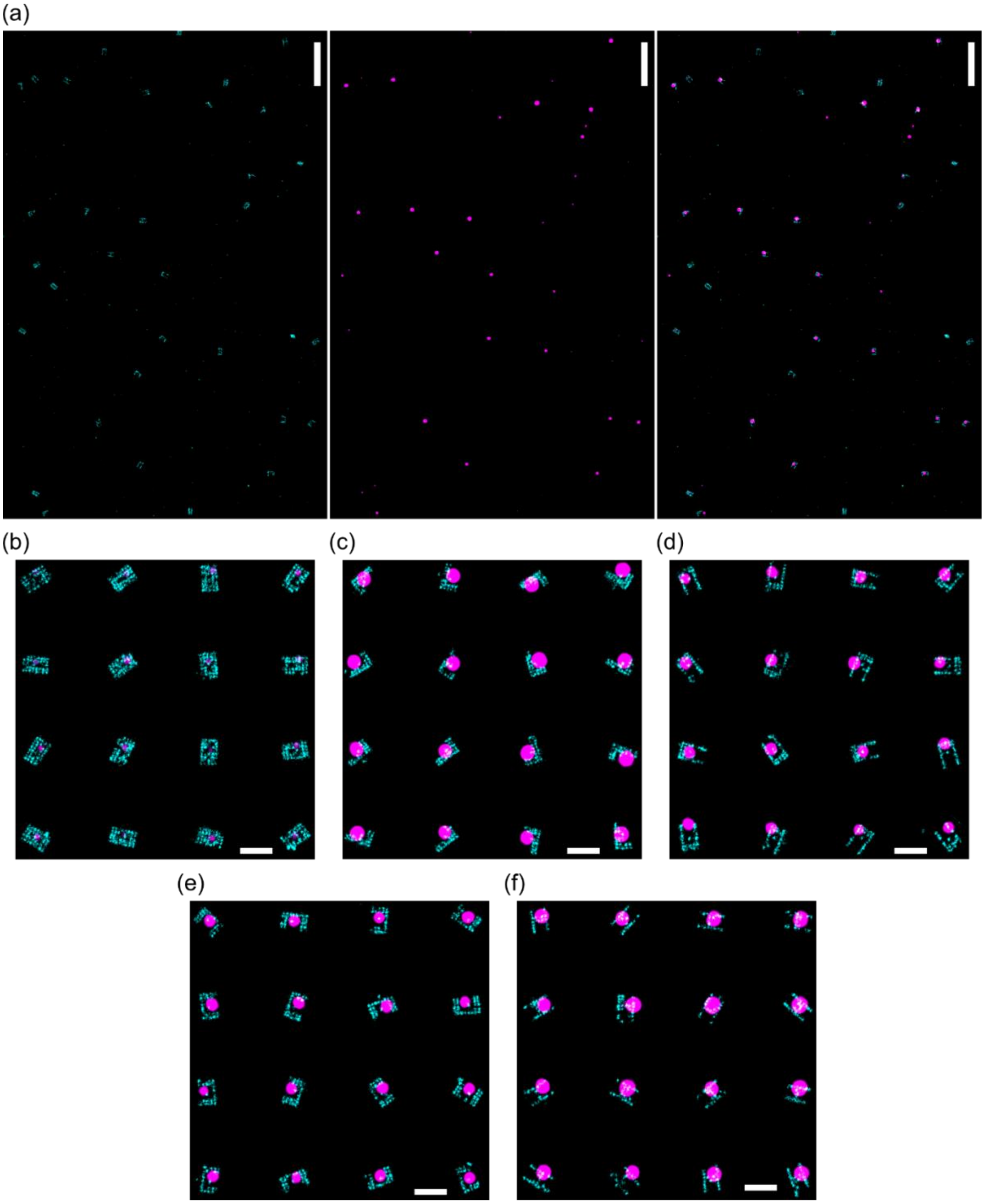

Supplementary Figure 2: DNA-PAINT images of simultaneous imaging showing the selected origami structures and corresponding assay site (a) Representative large field of view showing all five grid shapes. As mentioned earlier, we first image the grids with an imager strand carrying ATTO647N (left) following which we image the assay site with an imager strand carrying Cy3B (centre). These two channels are reconstructed individually and then merged (right) for manual picking and further analysis. (Scale = 500 nm); Montage of individually picked and aligned origami structures structures showing (b) box-shape for gap, (c) 'L'-shape for stem with A, (d) 'U'-shape for stem with T, (e) 'C'-shape for stem with C, (f) 'H'-shape for stem with G. (Scale Bar: 500 nm (a), 100 nm (b – f).

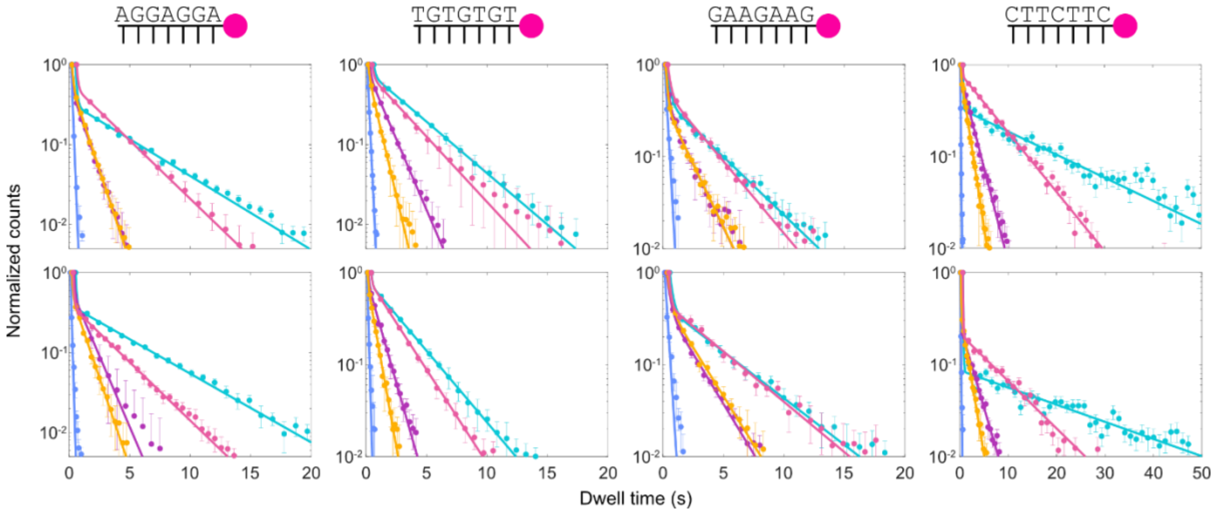

Supplementary Figure 3: Replicate-2 and replicate-3 data of figure (3a). Mono- and bi- exponential fits for individual dwell times under gap and different stackings, respectively. Color coding is the same as in (Supp Figure 4a). Error bars were generated via 100 iterations of bootstrapping. No. of dwell times analyzed for all data sets are shown in Extended Data Figure 2(a).

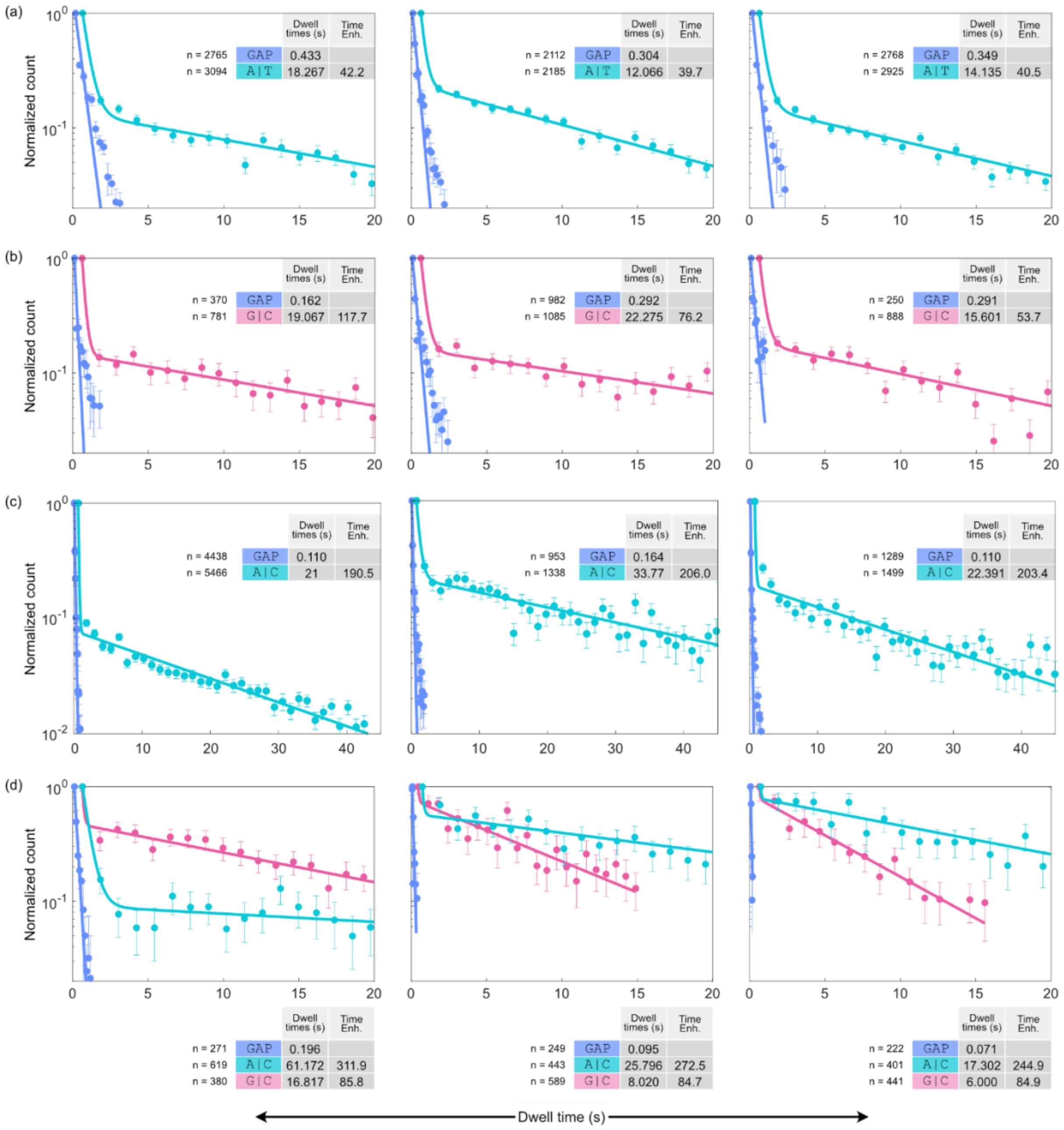

Supplementary Figure 4: (a) Mono- and bi- exponential fits of individual dwell times for the gap and A|T stack using a 9nt imager. This showed longer discrete binding times as compared to the 7nt imager but showed similar dwell time enhancements (b) Mono- and bi- exponential fits of individual dwell times for the gap and G|C stack using an 8nt imager. This showed longer discrete binding times as compared to the 7nt imager but showed similar dwell time enhancement. (c) Mono- and bi-exponential fits of individual dwell times of the gap and A|C stack, respectively, using orthogonal stem configuration as shown in Extended Data Figure 4a. Dwell times and enhancements show similar values under this orthogonal condition. (d) Mono- and bi- exponential fits of individual dwell times for the gap and nick configurations, respectively for Cy5-labeled imager. This showed longer discrete binding times as compared to imager containing Cy3B but showed similar dwell time enhancement.

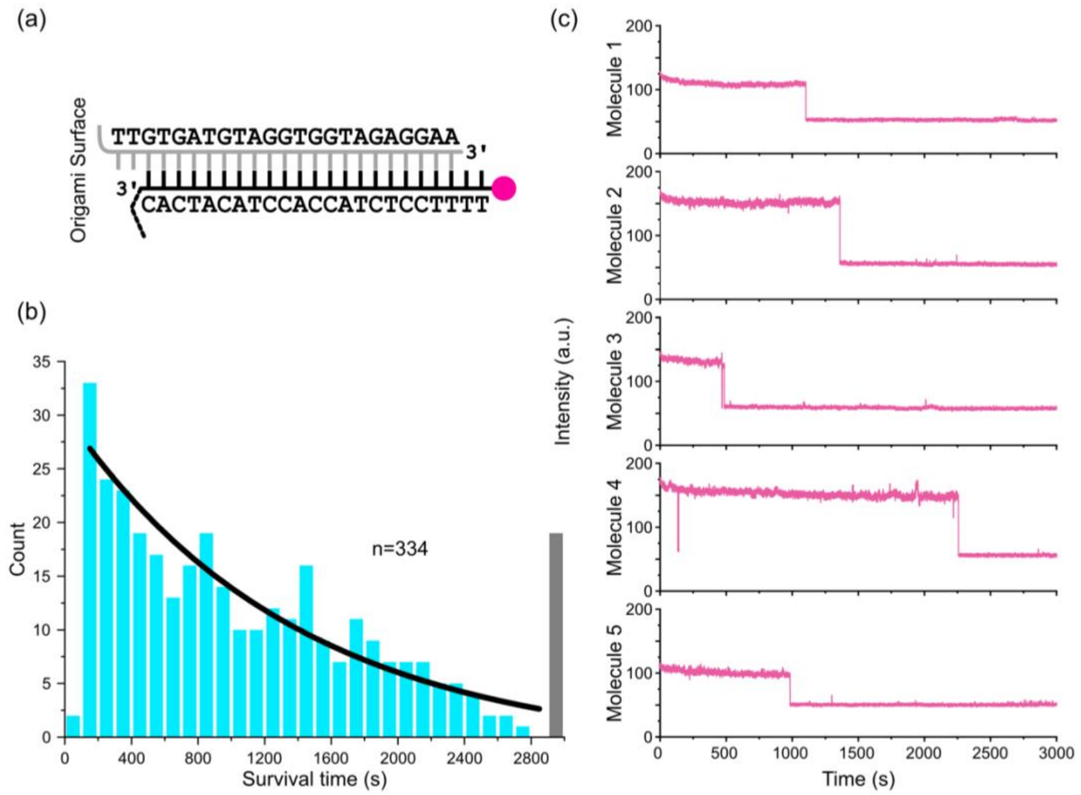

Supplementary Figure 5: (a) Graphical representation of the stable duplex used in the photobleaching experiment. (b) Plot showing number of Cy3B fluorophore molecules surviving for a given amount of time (n=313) before photobleaching. Single exponential decay fitting was performed on the histogram plot to obtain the bleaching rate of the fluorophore (0.0007/second). This is substantially lower than the obtained off rates for various stacks, ruling out any requirements of correction for photobleaching in the nick imaging studies. The first bar is ignored for the fitting. The colored bar represent the number of survived (fluorescing until the end of the imaging duration) molecules. (c) Intensity time traces for five individual molecules that showed single step fluorescence disappearance due to photobleaching.

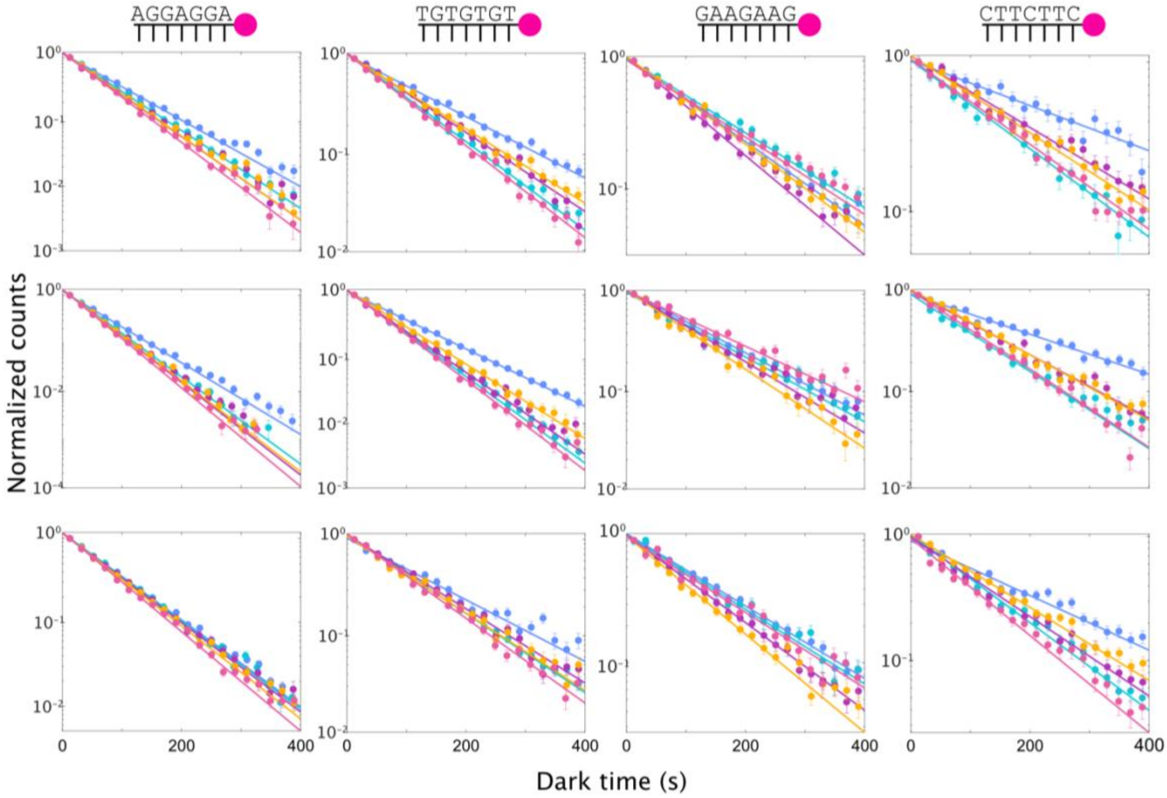

Supplementary Figure 6: (a) Histograms of dark time (points) fit with single exponential function (lines) of all four different imager sequences. Each row represents a replicate. Error bars were generated via 100 iterations of bootstrapping of at least 1000 individual dark times.

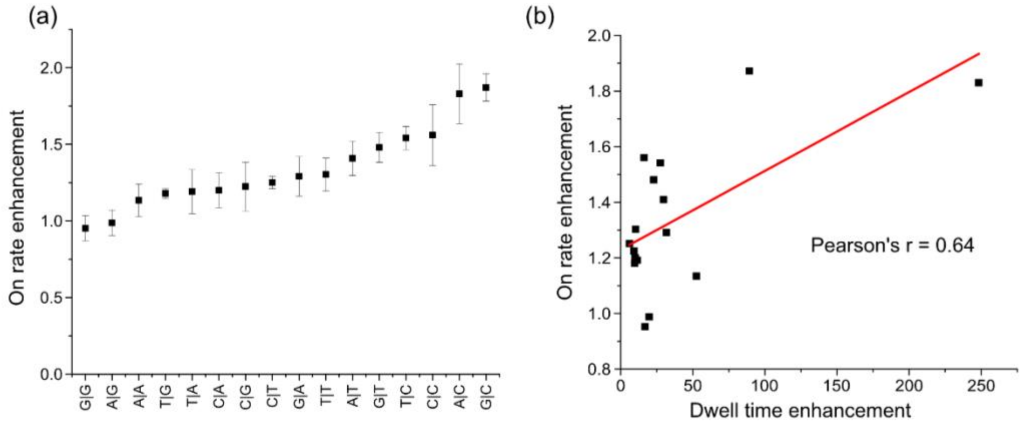

Supplementary Figure 7: (a) Plot showing correlation between the dwell time enhancements and the on-rate enhancements. There is a positive correlation between the dwell time enhancements and the on-rate enhancements. ($r^2 = 0.64$). Data represent means and standard deviations of three individual data sets. (b) Increase order of on-rate enhancement. Greater on-rate enhancements were clearly seen for stacks with pyrimidine|pyrimidine dinucleotides. Least on-rate enhancements were clearly noted in the case of stack with purine|purine dinucleotides. This would likely arise due to the bulky nature of the purines causing steric hindrance effects on imager binding.

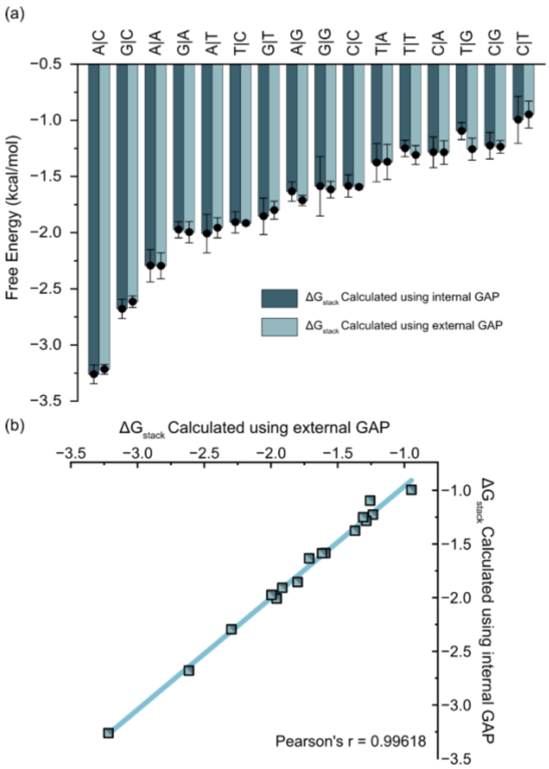

Supplementary Figure 8: (a) Plot showing free energies calculated from internal (first exponent of nick) and external gaps (control gap assay site). The two obtained values are similar. Data represent means and standard deviations of three individual data sets. (b) Correlation plot showing a high degree of similarity between the two calculated values thus ruling out any substantial effect of cross-stacking interactions.

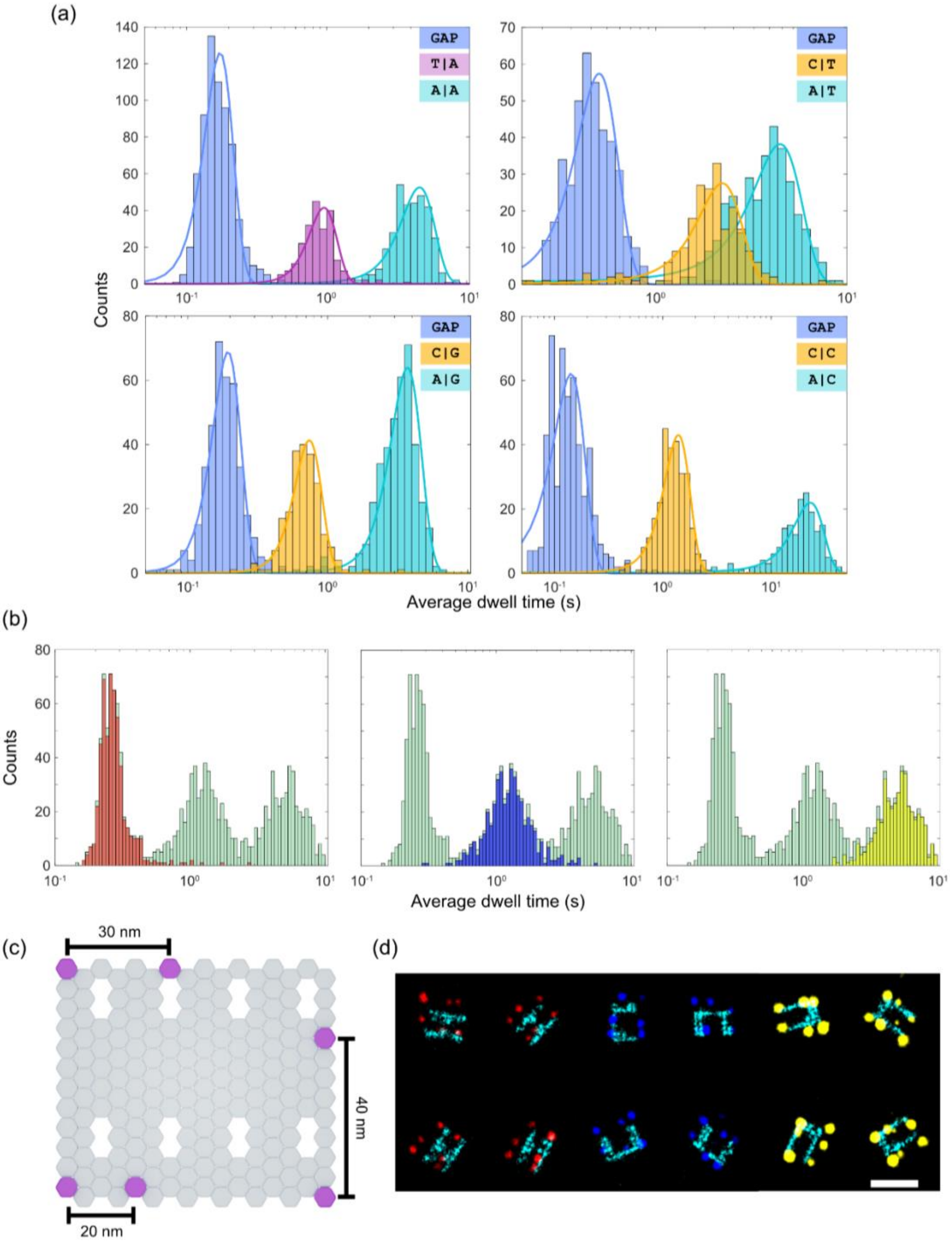

Supplementary Figure 9: Stack-PAINT for decoding of several targets with a single imager based on the varying average dwell times of the imager depending on the stacking partner. (a) Histograms showing the distribution of average dwell time for each of the four imagers used in figure 3a. Out of five configurations (a gap and four nicks), we show three potential pairs of stacking configurations that can be used for Stack-PAINT. (b) Manually picked origami based on the ground-truth grid structures show average binding times in three distinguishable peaks. Gap (red), T|A (blue), and A|A (yellow) show distinct average dwell time of the imager on the target. (c) Extensions on origami structures separated by varying distances used to measure the resolution potential of Stack-PAINT. (d) Picked origami structures showing the ground-truth grid structures along with the decoded target spots showing clear distinct 20 nm or farther separated spots with Stack-PAINT.

Supplementary table 1: Off rate constants resulted from the exponential fitting for the data sets in figure 3a, 3b, and supplementary figure 4. Off rate constants, obtained by taking the inverse of dissociation rate ($k_{off,2}$), and dwell time enhancement over gap for each replicate set.

	Stack	First rate constant (k_{off} or $k_{off,1}$)	Second rate constant ($k_{off,2}$)	Dwell time enhancement	Stack	First rate constant (k_{off} or $k_{off,1}$)	Second rate constant ($k_{off,2}$)	Dwell time enhancement
Replicate 1	**GAP**	6.915			**GAP**	9.919		
	**A|A**	7.137	0.122	56.417	**A|T**	11.900	0.297	33.359
	**T|A**	7.165	0.594	11.629	**T|T**	8.919	0.837	11.843
	**C|A**	7.165	0.659	10.493	**C|T**	11.920	1.431	6.929
	**G|A**	6.415	0.206	33.539	**G|T**	11.789	0.388	25.517
Replicate 2	**GAP**	8.866			**GAP**	7.435		
	**A|A**	8.366	0.212	41.717	**A|T**	6.935	0.296	25.057
	**T|A**	8.664	0.993	8.924	**T|T**	6.935	0.814	9.130
	**C|A**	8.366	1.056	8.393	**C|T**	6.935	1.480	5.022
	**G|A**	8.979	0.336	26.366	**G|T**	6.935	0.376	19.758
Replicate 3	**GAP**	11.854			**GAP**	11.064		
	**A|A**	12.064	0.198	59.83025	**A|T**	12.821	0.358	30.855
	**T|A**	12.069	0.816	14.52162	**T|T**	10.064	1.061	10.425
	**C|A**	12.104	1.030	11.508	**C|T**	12.231	1.711	6.465
	**G|A**	11.354	0.335	35.284	**G|T**	13.062	0.469	23.578
Replicate 1	**GAP**	4.056			**GAP**	12.072		
	**A|G**	3.726	0.191	21.212	**A|C**	12.538	0.048	247.329
	**T|G**	3.056	0.358	11.321	**T|C**	11.078	0.451	26.732
	**C|G**	3.919	0.408	9.935	**C|C**	11.072	0.736	16.382
	**G|G**	3.056	0.228	17.743	**G|C**	12.566	0.148	81.344
Replicate 2	**GAP**	5.333			**GAP**	13.357		
	**A|G**	4.333	0.290	18.352	**A|C**	13.779	0.057	230.371
	**T|G**	4.333	0.633	8.417	**T|C**	15.357	0.471	28.345
	**C|G**	6.753	0.632	8.433	**C|C**	15.357	0.794	16.808
	**G|G**	4.333	0.360	14.795	**G|C**	15.349	0.149	89.595
Replicate 3	**GAP**	4.667			**GAP**	11.482		
	**A|G**	4.244	0.233	20	**A|C**	13.436	0.043	267.486
	**T|G**	3.667	0.500	9.321	**T|C**	10.482	0.414	27.690
	**C|G**	3.667	0.486	9.593	**C|C**	10.482	0.730	15.724
	**G|G**	6.667	0.252	18.514	**G|C**	13.340	0.118	96.760

Supplementary table 2: Binding rate constants (k_{bind}) for all data sets shown in Supplementary figure 7 and enhancement in k_{bind} (i.e., ratio of stack to gap binding rates).

	Stack	Rate constant	Enhancement in binding rate	Stack	Rate constant	Enhancement in binding rate
Replicate 1	GAP	0.011		GAP	0.007	
	A|A	0.034	0.853	A|T	0.010	0.694
	T|A	0.014	0.797	T|T	0.009	0.776
	C|A	0.014	0.793	C|T	0.008	0.826
	G|A	0.016	0.738	G|T	0.011	0.667
Replicate 2	GAP	0.017		GAP	0.010	
	A|A	0.020	0.821	A|T	0.015	0.665
	T|A	0.022	0.772	T|T	0.014	0.706
	C|A	0.021	0.786	C|T	0.013	0.775
	G|A	0.023	0.726	G|T	0.016	0.637
Replicate 3	GAP	0.011		GAP	0.00	
	A|A	0.012	0.985	A|T	0.009	0.778
	T|A	0.012	0.973	T|T	0.008	0.830
	C|A	0.012	0.935	C|T	0.009	0.797
	G|A	0.013	0.875	G|T	0.010	0.727
Replicate 1	GAP	0.007		GAP	0.003	
	A|G	0.006	1.119	A|C	0.006	0.510
	T|G	0.008	0.845	T|C	0.005	0.631
	C|G	0.007	0.954	C|C	0.005	0.583
	G|G	0.006	1.086	G|C	0.006	0.529
Replicate 2	GAP	0.007		GAP	0.004	
	A|G	0.007	0.961	A|C	0.009	0.520
	T|G	0.008	0.873	T|C	0.007	0.630
	C|G	0.009	0.788	C|C	0.007	0.613
	G|G	0.006	1.121	G|C	0.009	0.512
Replicate 3	GAP	0.006		GAP	0.005	
	A|G	0.007	0.970	A|C	0.008	0.622
	T|G	0.008	0.825	T|C	0.007	0.688
	C|G	0.009	0.736	C|C	0.006	0.748
	G|G	0.007	0.956	G|C	0.009	0.563

Supplementary table 3: ΔG_{stack} comparison from different studies. Columns two, three and four are free energy of base-pair stacking interactions extracted from unified model for the nearest-neighbor parameters[1], gel migrated DNA with nick and gap configurations (37 °C)[2], and blunt end interactions of DNA bundles extrapolated to zero-force and single-pair base-stacks from optical tweezers experiments (Room temperature)[3], respectively. We have taken these numbers from Zacharias [4]. Column five outlines base-stacking energetics obtained from a study involving DNA micro-chips (measurements interpreted at 37 °C).[5] Column six tabulates the base stacking energies obtained from thermal denaturation measurements of stem looped DNA (measurements interpreted at 37 °C).[6] Column seven is the free energy calculation of individual dinucleotide base-stacking interactions from the current study. The eighth column shows the average of reverse complements of dinucleotide base-stacks from this study.

Base-pair	$\Delta G_{,SantaLucia}$ (kcal.mol^{-1})	$\Delta G_{,Yakovchuk}$ (kcal.mol^{-1})	$\Delta G_{,Kilchherr}$ (kcal.mol^{-1})	$\Delta G_{,Vasiliskov}$ (kcal.mol^{-1})	$\Delta G_{,Pyshnyi}$ (kcal.mol^{-1})	ΔG (kcal.mol^{-1})	ΔG_{avg} (kcal.mol^{-1})
A|A, T|T	−1.00	−1.11	−1.36	-1.8, -0.8	-1.93, -1.22	-2.30, -1.31	-1.80
AT	−0.88	−1.34	−2.35	-1.4	-1.89	-1.96	-1.96
TA	−0.58	−0.19	−1.01	-0.8	-0.85	-1.37	-1.37
G|G, C|C	−1.84	−1.44	−1.64	-0.8, -0.7	-1.96, -1.25	-1.62, -1.60	-1.60
GC	−2.24	−2.17	−3.42	-0.9	-2.76	-2.61	-2.61
CG	−2.17	−0.91	−2.06	-0.5	-1.17	-1.24	-1.24
T|G, C|A	−1.45	−0.55	−0.81	-0.7, -0.8	-1.2, -1.06	-1.26, -1.29	-1.27
A|G, C|T	−1.28	−1.06	−1.60	-1.6, -0.5	-1.35, -1.19	-1.71, -0.95	-1.33
T|C, G|A	−1.30	−1.43	−1.39	-1.2, -1.0	-1.46, 2.21	-1.92, -2.00	-1.96
G|T, A|C	−1.44	−1.81	−2.03	-1.0, -1.95	-2.1, -2.29	-1.80, -3.22	-2.51

Supplementary table 4: Off rate constants resulted from the exponential fitting for the base-modifications data sets in figure 3d-f. Off rate constants, obtained by taking the inverse of dissociation rate ($k_{off,2}$), and dwell time enhancement over gap for each replicate set.

		Stack	First rate constant (k_{off} or $k_{off,1}$)	Second rate constant ($k_{off,2}$)	Dwell time enhancement
Fluorophore Modification	Rep 1	GAP	7.873		
		A|Cy3B|C	6.873	0.491	16.035
	Rep 2	GAP	8.827		
		A|Cy3B|C	7.827	0.636	13.880
	Rep3	GAP	7.037		
		A|Cy3B|C	6.037	0.330	21.344
meC|A	Rep 1	GAP	20.775		
		meC|A	19.775	1.909	10.884
	Rep 2	GAP	13.357		
		meC|A	15.357	1.848	7.226
	Rep3	GAP	9.280		
		meC|A	11.280	1.826	5.083
meC|C	Rep 1	GAP	10.443		
		meC|C	9.443	0.878	11.892
	Rep 2	GAP	11.382		
		meC|C	13.382	0.885	12.861

	Rep3	**GAP**	13.342		
		**meC|C**	12.342	1.026	12.998
meC|G	Rep 1	**GAP**	6.312		
		**meC|G**	6.575	0.648	9.734
	Rep 2	**GAP**	7.236		
		**meC|G**	9.236	0.873	8.290
	Rep3	**GAP**	8.275		
		**meC|G**	7.276	0.695	11.907
meC|T	Rep 1	**GAP**	12.531		
		**meC|T**	14.531	1.947	6.436
	Rep 2	**GAP**	15.143		
		**meC|T**	17.143	1.837	8.244
	Rep3	**GAP**	15.629		
		**meC|T**	14.630	2.000	7.815
Inosine|Adenine	Rep 1	**GAP**	4.760		
		**C-I|A**	3.760	0.845	5.635
		**A-I|A**	5.451	1.025	4.645
	Rep 2	**GAP**	4.676		
		**C-I|A**	4.255	0.667	7.015
		**A-I|A**	4.342	1.037	4.510
	Rep 3	**GAP**	6.893		
		**C-I|A**	7.782	1.188	5.802
		**A-I|A**	8.894	2.000	3.447
Inosine|Guanine	Rep 1	**GAP**	8.593		
		**C-I|G**	7.593	2.000	4.297
		**A-I|G**	7.593	2.000	4.29664
	Rep 2	**GAP**	5.568		
		**C-I|G**	7.568	1.514	3.678
		**A-I|G**	7.568	1.807	3.082
	Rep 3	**GAP**	5.334		
		**C-I|G**	4.572	0.909	5.868
		**A-I|G**	7.334	1.082	4.929

Supplementary table 5: Sequences used for Tetrahedron Folding

Staple Name	Staple Sequence	Manufacturer
L	AGGCACCATCGTAGGTTTTTCTTGCCAGGCACCATCGTAGGTTTTTCTTGCCAGGCACCATCGTAGGTTTTTCTTGCC	Integrated DNA Technologies
M_AC	CTGCAACCTGCCTGGCAAGCCTACGATGGACACGGTAGTCGA	Integrated DNA Technologies
S_AC	CTACCGTGTGGTTGCAGTCGA	Integrated DNA Technologies
M_GA	AGGCAACCTGCCTGGCAAGCCTACGATGGACACGGTCTCTAG	Integrated DNA Technologies
S_GA	AGACCGTGTGGTTGCCTCTAG	Integrated DNA Technologies
M_CT	TGGCAACCTGCCTGGCAAGCCTACGATGGACACGGTCAGTAC	Integrated DNA Technologies
S_CT	TGACCGTGTGGTTGCCAGTAC	Integrated DNA Technologies
S_AC_BLK	TACCGTGTGGTTGCAGTCGA	Integrated DNA Technologies
S_GA_BLK	GACCGTGTGGTTGCCTCTAG	Integrated DNA Technologies
S_CT_BLK	GACCGTGTGGTTGCCAGTAC	Integrated DNA Technologies

Supplementary table 6: Sequences used for different tetrahedrons.

Origami Name	Sequences used in 1:3:3 ratio
2×AC	L + M_AC + S_AC
2×GA	L + M_GA + S_GA
2×CT	L + M_CT + S_CT
1×AC	L + M_AC + S_AC_BLK
1×GA	L + M_GA + S_GA_BLK
1×CT	L + M_CT + S_CT_BLK

Supplementary table 7: Imaging Parameters

Figure Number/ Data Sets	Laser Power (W/cm^2)		Imager Concentrations (nM)		Parameters (Exposure time, number of frames)	
	561 nm	640 nm	Cy3B	Atto647N	561nm	640nm
Figure 1d Left; Supp Figure-1a Left	26	490	3	0.5	50 ms, 50000	100ms, 25000
Figure 1d Right; Supp Figure-1a Right	26	852	3	0.5	50 ms, 50000	100ms, 25000
Figure 2 b, c; Supp Fig 2	26	490	3	0.5	50 ms, 50000	100ms, 25000
Figure 3a(i); Ext Data Figure-2a (Replicate-1,3); Supp Figure-3 (Row 2); Supp Figure 5 (Replicate 1, 3) (Imagers: AGGAGGA-Cy3B, GAAGAAG-Atto647N)	26	490	2.5	0.5	50 ms, 50000	100ms, 25000
Figure 3a(iii); Ext Data Figure-2a (Replicate 1, 3); Supp Figure-3 (Row 2); Supp Figure 5 (Replicate 1, 3) (Imagers: TGTGTGT-Cy3B, AGGAGGA-Atto647N)	26	490	2.5	0.5	50 ms, 50000	100ms, 25000
Figure 3a(ii); Ext Data Figure-2a; Supp Figure-3; Supp Figure 5 (Imagers: GAAGAAG-Cy3B; AGGAGGA-Atto647N)	26	490	3	0.5	50 ms, 50000	100ms, 25000
Figure 3a(iv); Ext Data Figure-2a; Supp Figure-3; Supp Figure 5 (Imagers: CTTCTTC-Cy3B; AGGAGGA-Atto647N)	26	490	2.5	0.5	50 ms, 50000	100ms, 25000
Ext Data Figure 2a (Replicate 2); Supp Figure-3 (Row 1); Supp Figure 5 (Replicate 2) (Imagers: AGGAGGA-Cy3B, GAAGAAG-Atto647N)	26	490	3	0.5	50 ms, 50000	100ms, 25000
Ext Data Figure 2a (Replicate 2); Supp Figure-3 (Row 1); Supp Figure 5 (Replicate 2) (Imagers: TGTGTGT-Cy3B, AGGAGGA-Atto647N)	26	490	5	0.5	50 ms, 50000	100ms, 25000
Supp Figure 4c (Imagers: CTTCTTC-Cy3B; AGGAGGA-Cy3B, TGTGTGT-Cy3B)	26	490	2.5, 0.5, 0.5 respectively		50 ms, 50000	100ms, 10000
Supp Figure 4d (Imagers: CTTCTTC-Cy5; AGGAGGA-Cy3B)	313	57	0.5	1.5 (Cy5)	100 ms, 25000	50 ms, 50000
Supp Figure 4 b, c (Stable Binder: Cy3B-TTTTCCTCTACCACCTACATCACTTACCACCACCACCA	26				200 ms, 15000	
Figure 4(f-i), Supp Figure 9 (Imagers: AGGAGGA-Cy3B, GAAGAAG-Atto647N)	104	490	0.5	0.5	50ms, 72000	100ms, 25000
Figure 3d (Imagers: AGGAGGA-Cy3B, GAAGAAG-Atto647N)	26	490	2 and 3	0.5	50ms, 50000	100ms, 25000
Figure 3e (Imagers: meC|A: AGGAGGA-Cy3B, GAAGAAG-Atto647N)	26	490	2	0.5	50ms, 50000	100ms, 25000
Figure 3e (Imagers: meC|C: CAACAAC-Cy3B, AGGAGGA-Atto647N)	26	490	2	0.5	50ms, 50000	100ms, 25000
Figure 3e (Imagers: meC|G: GAAGAAG-Cy3B, AGGAGGA-Atto647N	26	490	2	0.5	50ms, 50000	100ms, 25000
Figure 3e (Imagers: meC|T: TGTGTGT-Cy3B, AGGAGGA-Atto647N)	26	490	2	0.5	50ms, 50000	100ms, 25000

Figure 3f (Imagers: C-I|A and A-I|A: AGGAGGA-Cy3B, GAAGAAG-Atto647N; C-I|G and A-I|G: GAAGAAG-Cy3B, AGGAGGA-Atto647N)	26	490	2 and 3 respectively.	0.5	50ms, 50000	100ms, 25000
Supp Figure 4b (Imagers: CAACAACA-Cy3B, AGGAGGA-Atto647N)	26	490	1 and 2	0.5	50ms, 50000	100ms, 25000
Supp Figure 4a (Imagers: TAGATGTAT-Cy3B, AGGAGGA-Atto647N)	26	490	4	0.5	50ms, 50000	100ms, 25000

References

1 SantaLucia Jr, J. A unified view of polymer, dumbbell, and oligonucleotide DNA nearest-neighbor thermodynamics. *Proceedings of the National Academy of Sciences* **95**, 1460-1465 (1998).

2 Yakovchuk, P., Protozanova, E. & Frank-Kamenetskii, M. D. Base-stacking and base-pairing contributions into thermal stability of the DNA double helix. *Nucleic Acids Research* **34**, 564-574, doi:10.1093/nar/gkj454 (2006).

3 Kilchherr, F. *et al.* Single-molecule dissection of stacking forces in DNA. *Science* **353**, aaf5508, doi:doi:10.1126/science.aaf5508 (2016).

4 Zacharias, M. Base-Pairing and Base-Stacking Contributions to Double-Stranded DNA Formation. *The Journal of Physical Chemistry B* **124**, 10345-10352, doi:10.1021/acs.jpcb.0c07670 (2020).

5 Vasiliskov, V. A., Prokopenko, D. V. & Mirzabekov, A. D. Parallel multiplex thermodynamic analysis of coaxial base stacking in DNA duplexes by oligodeoxyribonucleotide microchips. *Nucleic Acids Research* **29**, 2303-2313, doi:10.1093/nar/29.11.2303 (2001).

6 Pyshnyi, D. V. *et al.* Thermodynamic Analysis of Stacking Hybridization of Oligonucleotides with DNA Template. *Journal of Biomolecular Structure and Dynamics* **19**, 555-570, doi:10.1080/07391102.2001.10506763 (2001).
