# Supplementary material for: Single-molecule analysis of DNA base-stacking energetics using patterned DNA nanostructures
Source: Nat Nanotechnol. 2023 Aug 17;18(12):1474–82. doi: 10.1038/s41565-023-01485-1 (PMC10716042; doi:10.1038/s41565-023-01485-1)

Uncropped gel Images: Uncropped gel images used for measuring the degree of assembly of Multimeric Tetrahedral origami structures. (a-c) show gels with 2× stacking interactions and (d-f) show gels with 1× stacking interactions. Red boxes represent the regions displayed in the Main Figures and Extended Data Figures. Samples in other lanes are not relevant to the current study.

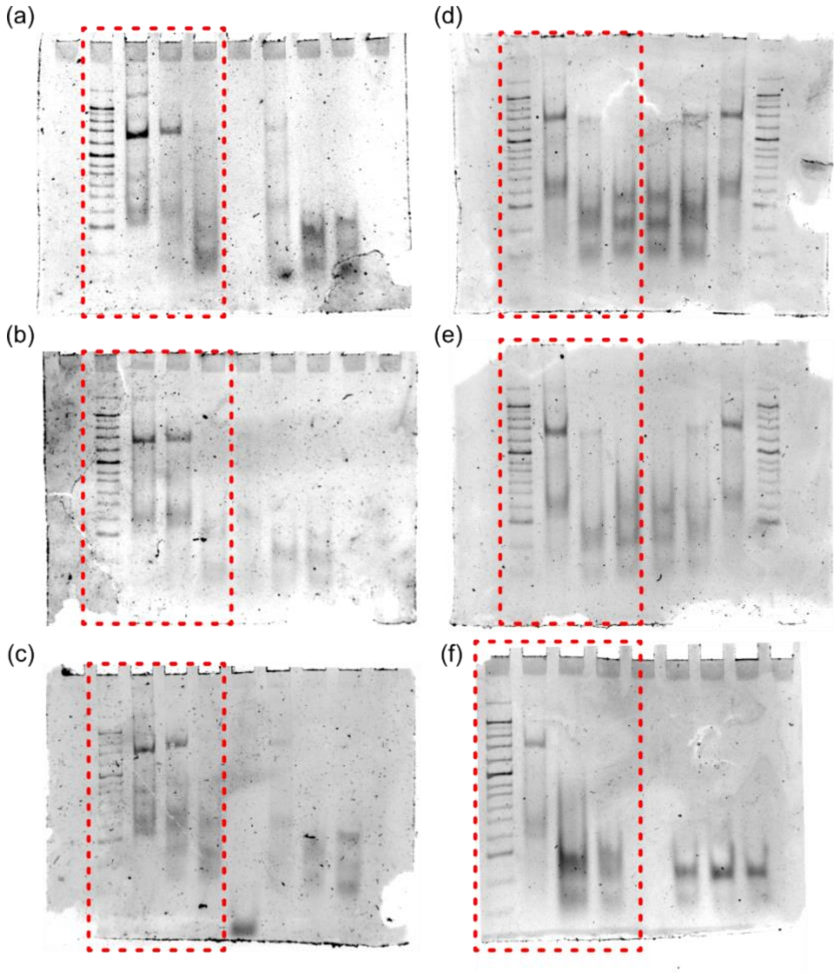

Supplement: Supplementary file 11 — Uncropped gel images. [file 41565_2023_1485_MOESM11_ESM.pdf]
